# Mass spectrometry reveals potential of β-lactams as SARS-CoV-2 M^pro^ inhibitors†
†Electronic supplementary information (ESI) available: Experimental details and inhibition data. See DOI: 10.1039/d0cc06870e


**DOI:** 10.1039/d0cc06870e

**Published:** 2021-01-19

**Authors:** Tika R. Malla, Anthony Tumber, Tobias John, Lennart Brewitz, Claire Strain-Damerell, C David Owen, Petra Lukacik, H. T. Henry Chan, Pratheesh Maheswaran, Eidarus Salah, Fernanda Duarte, Haitao Yang, Zihe Rao, Martin A. Walsh, Christopher J. Schofield

**Affiliations:** a Chemistry Research Laboratory , Department of Chemistry , 12 Mansfield Road , Oxford , OX1 3TA , UK . Email: christopher.schofield@chem.ox.ac.uk; b Diamond Light Source , Harwell Science & Innovation Campus , Didcot , Oxfordshire OX11 0DE , UK; c Research Complex at Harwell , Harwell Science & Innovation Campus , Didcot , Oxfordshire OX11 0FA , UK; d Shanghai Institute for Advanced Immunochemical Studies and School of Life Science and Technology , ShanghaiTech University , Shanghai , China

## Abstract

The main viral protease (M^pro^) of SARS-CoV-2 is a nucleophilic cysteine hydrolase and a current target for anti-viral chemotherapy. We describe a high-throughput solid phase extraction coupled to mass spectrometry M^pro^ assay. The results reveal some β-lactams, including penicillin esters, are active site reacting M^pro^ inhibitors, thus highlighting the potential of acylating agents for M^pro^ inhibition.

## Abstract 2

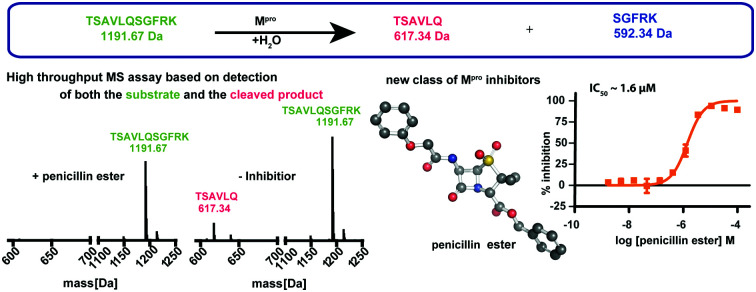
A high-throughput mass spectrometry based M^pro^ assay identifies penicillin esters as new SARS-CoV-2 M^pro^ inhibitors.

## 

The main viral protease (M^pro^) of severe acute respiratory syndrome coronavirus 2 (SARS-CoV-2)^1^ is a COVID-19 treatment target.^2^ M^pro^ along with the papain-like protease (PL^pro^), processes initially translated viral polyproteins to give cleaved proteins with biological functions essential for viral replication in cells.^3^ Following formation of a non-covalent enzyme-substrate complex, M^pro^ catalysis proceeds *via* His-41 enabled reaction of Cys-145 with a scissile peptide bond forming a hydrolytically labile thioester. M^pro^ cleaves after glutamine-residues with a preference for small-residues on the C-terminal side of the cleaved amide (Fig. 1A and B).^4^

**Fig. 1:**
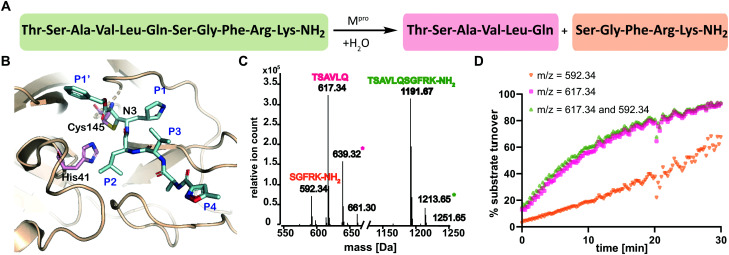
SPE-MS assay monitoring M^pro^ catalyzed cleavage of the TSAVLQ/SGFRK-NH_2_ substrate. (A) M^pro^ catalyzed hydrolysis of TSAVLQ/SGFRK-NH_2_. (B) View from a structure of Cys-145 linked M^pro^-N3 complex (**PDB ID: 6LU7**);^1^ the Cys–His dyad is in pink; substrate binding sites are labelled in blue. (C) Deconvoluted mass spectrum of substrate/cleaved products after 10 minute incubation with M^pro^. Note, the C-terminal product was not efficiently retained by the SPE cartridge resulting in a low abundance compared to the N-terminal cleavage product. Sodium ion adducts (+23 Da) for the TSAVLQ product (639 Da) and substrate (1214 Da) are labelled with magenta and green asterisks, respectively. (D) % substrate turnover based on integration of the total abundance of cleaved products (TSAVLQ or SGFRK-NH_2_) or the individual products. Conditions: 0.15 μM M^pro^, 2.0 μM TSAVLQ/SGFRK-NH_2_ (1192 Da) (20 mM HEPES, pH 7.5, 50 mM NaCl).

Most reported M^pro^ assays measure fluorescence, as precedented for other protease assays.^1^,2b,^5^ Whilst efficient, such methods do not simultaneously monitor substrate depletion/product formation and some compounds interfere with fluorescence.^6^ We were thus interested in establishing an alternative mass spectrometry (MS)-based high-throughput M^pro^ assay for identifying new inhibitors and testing known drugs.

Solid phase extraction coupled with MS (SPE-MS) has been applied to high-throughput screens of enzymes.^7^ We envisaged it could simultaneously monitor both M^pro^ substrate depletion/product formation and covalent modification. The latter is of interest because many reported inhibitors of nucleophilic cysteine enzymes work by covalent reaction.^8^ Here we report how such an assay enabled identification of new M^pro^ inhibitors, including β-lactams, the most important antibacterial class.^9^

We developed conditions for an SPE-MS based SARS-CoV-2 M^pro^ assay (0.15 μM M^pro^, 2.0 μM TSAVLQ/SGFRK-NH_2_, 20 mM HEPES, pH 7.5, 50 mM NaCl) using protein prepared as reported.^1^,^10^ Isolated M^pro^ was found to be active when monitoring turnover of peptide substrates, including TSAVLQ/SGFRK-NH_2_ which was cleaved to give TSAVLQ and SGFRK-NH_2_ fragments (Fig. 1C and D). Kinetic parameters were determined for the 11-mer substrate (*K*_m_ = 14.4 μM; *k*_cat_ = 2.7 min^–1^), both by monitoring substrate depletion and N-terminal product fragment formation (Fig. S1, ESI†). The efficiency (*k*_cat_*/K*_m_) of M^pro^ determined by SPE-MS (28 500 M^–1^ s^–1^) is comparable to that observed for a similar substrate Mca–AVLQ/SGFRK(Dnp)K using a fluorescence resonance energy transfer (FRET) assay (27 000 M^–1^ s^–1^, as reported and in our hands).^1^ Steady state kinetics for a 37-mer substrate were also investigated; a 2-fold increase in *k*_cat_*/K*_m_ (60 026 M^–1^ s^–1^) was observed (Fig. S2, ESI†). Comparison of kinetic parameters for the SARS-CoV-2 M^pro^ and the related SARS-CoV M^pro^ reveal similar *k*_cat_/*K*_m_ values (though the values for SARS-CoV were somewhat lower when using shorter substrates in an HPLC assay) (Table S1, ESI†). Note, the interconversion between monomeric/dimeric forms of M^pro^ has the potential to introduce complexity in kinetic analyses.^11^

Next, the SPE-MS assay was validated for inhibition studies with ebselen,^1^ N3,^1^ disulfiram,^1^ and boceprevir^12^ using the 11-mer TSAVLQ/SGFRK-NH_2_ substrate (Table 1 and Fig. S3, ESI†). The ebselen IC_50_ was ∼0.09 μM under standard conditions (0.15 μM M^pro^, 2.0 μM TSAVLQ/SGFRK-NH_2_*i.e.* [S] < *K*_m_, 20 mM HEPES, pH 7.5, 50 mM NaCl at ambient temperature) compared to an IC_50_ of ∼0.67 μM^1^ using a FRET assay (0.2 μM M^pro^, 20 μM Mca–AVLQ/SGFRK(Dnp)K *i.e.* [S] ≈ *K*_m_, 50 mM Tris–HCl, pH 7.3, 1 mM EDTA, 30 °C)^1^ (Table 1, entry 1; Fig. S3D, ESI†).

**Table 1:** IC_50_s of selected M^pro^ inhibitors determined using SPE-MS assays compared to those obtained using FRET assays

Inhibitor	IC_50_ (SPE-MS) [μM]^*a*^ ^*b*^	IC_50_ (SPE-MS)^*a*^ ^*c*^ [μM]	IC_50_ (FRET) [μM]
Ebselen	0.09 ± 0.07	0.09 ± 0.07	0.67 ± 0.09^1^
N3	0.04 ± 0.01	0.03 ± 0.01	n.d.
Disulfiram	0.60 ± 0.01	0.46 ± 0.02	9.35 ± 0.18^1^
Boceprevir	11.0 ± 4.8	9.2 ± 5.5	2.70 ± 0.05^12^

^*a*^Mean of two independent replicates each performed in technical duplicate (*n* = 2 ± standard deviation, SD). Conditions: 0.15 μM M^pro^ and 2.0 μM TSAVLQ/SGFRK-NH_2_ substrate in 20 mM HEPES, pH 7.5, 50 mM NaCl.

^*b*^30 min inhibitor preincubation.

^*c*^60 min inhibitor preincubation.

We optimized the assay for studying covalent modifications with a higher M^pro^ concentration being used to enable robust analyses (1 μM M^pro^), though IC_50_ and preliminary covalent modification data can be accumulated from the same experiment. SPE purification is denaturing, so monomer modification was observed. Assay validation used N3;^1^ predominantly (but not exclusively) a single N3 adduct was observed (Fig. 2A, B and Fig. S4, ESI†), consistent with structural work revealing Cys-145 reaction^1^ (Fig. 1B and Fig. S5, Table S2, ESI†). We exploited selective reaction of N3 to test selectivity of other inhibitors as exemplified with ebselen, comparing results for N3 treated/untreated M^pro^ (Fig. 2C, E and Fig, S6, ESI†). By contrast with N3, we saw time dependent modification of multiple residues with ebselen with or without N3 pre-treatment (Fig. 2C and E), implying reaction of some of the 11 non-active site cysteines (Fig. S5, ESI†). Ebselen was used as a readily available positive inhibition control in subsequent studies.

**Fig. 2:**
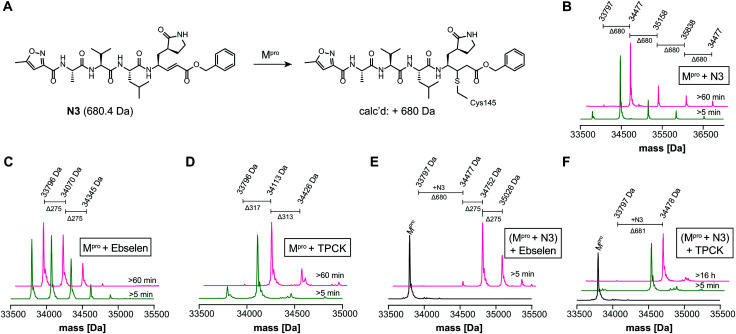
N3 dependent reaction monitoring the active site selectivity of inhibitors. (A) Reaction of N3 with the M^pro^ active site Cys-145.^1^ (B) N3, (C) ebselen and (D) TPCK modifies M^pro^ in a covalent manner. (E) Ebselen covalently modifies multiple M^pro^ cysteine residues in the presence of N3. (F) TPCK does not covalently modify M^pro^ in the presence of N3, suggesting that it selectively reacts with active site Cys-145. Black spectra: wild-type M^pro^ (33796 Da).

The assay was used to screen the Library of Pharmacologically Active Compounds (LOPAC) and a library of 1600 small-molecule active pharmaceutical ingredients (API) at 20 μM compound (Fig. S7, ESI†). Excellent Z′-factors^13^ and signal to noise ratios reveal the assay robustness (Fig. S7, ESI†). In addition to ebselen (identification of which validates the method), diverse inhibitors (≥80% at a fixed 20 μM inhibitor concentration) were identified, some (related to) known inhibitors,^1^,^14^ including auranofin, cisplatin, IPA-3, bismuth subsalicylate, thioguanine, carmustine, and disulfiram (Tables S3 and S4, ESI†).

IC_50_s were determined for compounds with ≥80% inhibition at 20 μM, excluding known interference compounds.^15^ Auranofin (IC_50_ ∼ 1.5 μM; reported IC_50_ ∼ 0.5 μM 14), an α-chloroketone (TPCK) (IC_50_ ∼ 0.8 μM), IPA-3 (IC_50_ ∼ 0.1 μM), and 5-thioguanine (IC_50_ ∼ 13.5 μM) are some of the more potent inhibitors (Fig. S8, ESI†). Some of these covalently modified M^pro^, sometimes with more than one reaction being observed (Fig. S9–S15, ESI†). Active site selectivity was investigated using N3 treated and untreated M^pro^. Following N3 treatment, in some cases, *e.g.* TPCK and N_α_-*p*-toluenesulfonyl-l-lysine chloromethyl ketone, substantial covalent modification was no longer observed, implying selective Cys-145 reaction (Fig. 2D, F and Fig. S12, S13, ESI†). Although further validation is required, with BAY 11-7082 and IPA-3 the multiple adducts observed with unmodified M^pro^ were diminished when the active site was N3 blocked, suggesting reaction with Cys-145 might alter the M^pro^ conformation (Fig. S9 and S11, ESI†).

The screen identified β-lactam drugs as potential M^pro^ inhibitors, including penicillins and cephalosporins (Table S5, ESI†). This was of interest, as in preliminary work we observed some β-lactams react covalently (data not shown). β-Lactam antibiotics form stable acyl–enzyme complexes with bacterial nucleophilic serine enzymes; they inhibit other nucleophilic serine enzymes including proteases and β-lactamases^16^ and nucleophilic cysteine enzymes.^17^

Studies on cephalosporins identified as potential inhibitors from the screen revealed no substantial covalent M^pro^ modification, though cephalosporin C Zn(ii) salt and cephalosporin C Na(i) salt inhibited. However, the IC_50_s for cephalosporin C Zn(ii) salt and ZnCl_2_ were similar, indicating much of the inhibition is due to Zn(II) ions (Fig. S16, ESI†), as observed for cephalosporin C Zn(ii) salt inhibition of other enzymes.7*b*

We further investigated β-lactam reactions with M^pro^ using a diverse set of β-lactams (Fig. S17, ESI†). Though most β-lactams were inactive (IC_50_ > 100 μM), two penicillin esters manifested IC_50_s < 5 μM, *i.e.***1**: a penicillin V sulfone C3 benzyl ester (IC_50_ ∼ 1.5 μM), and **2**: a derivative of penicillin G sulfoxide C3 *p*-nitrobenzyl ester (IC_50_ ∼ 3.5 μM), both with similar potency with either 30 or 60 min preincubation (Fig. 3A and B). Other β-lactams inhibited, though more weakly (Fig. S17, ESI†). The inhibition by the penicillin benzyl esters may, in part, reflect binding of the N3 benzyl ester, likely binding in the P1′ or P2 pocket (Fig. 1B).^1^ Structures of M^pro^ complexed with a β-lactam were not obtained; however, docking studies reveal potential of **1** and **2** to bind favourably at the active site (Fig. S18, ESI†), in the case of **1** in a manner enabling Cys-145 reaction.

**Fig. 3:**
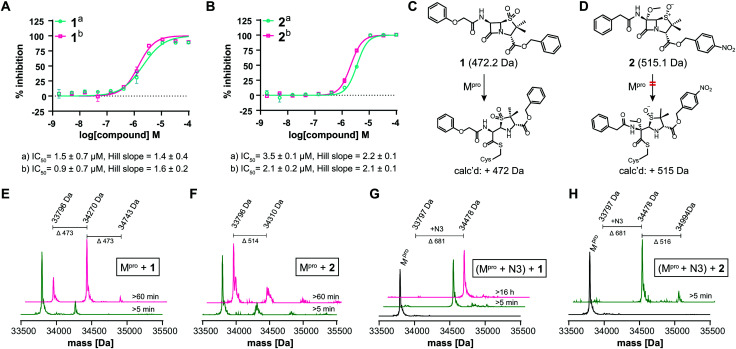
β-Lactams inhibit M^pro^. IC_50_s for (A) **1** (penicillin V sulfone C3 benzyl ester) and (B) **2** (C6-methoxy penicillin G sulfoxide C3 p-nitrobenzyl ester) determined using SPE-MS; data are a mean of technical duplicates with (a) 30 min and (b) 60 min preincubation. IC_50_s are means of two independent repeats each composed of technical duplicates (*n* = 2 ± SD). Proposed reaction of **1** (C) and **2** (D) with M^pro^. (E) A single molecule **1** covalently modifies M^pro^. (F) **2** does not efficiently modify M^pro^ through covalent reaction. Conditions: 1 μM M^pro^, 20 μM β-lactam, 20 mM HEPES, pH 7.5, 50 mM NaCl. (G) **1** does not covalently modify M^pro^ preincubated with N3, suggesting **1** reacts with Cys-145. (H) **2** does not efficiently react with M^pro^ preincubated N3. Conditions: 1 μM M^pro^ preincubated with 3 μM N3, 20 μM **1** or **2**, 20 mM HEPES, pH 7.5, 50 mM NaCl. Black spectra: wild-type M^pro^ (33 796 Da).

Evidence for covalent reaction was observed with representatives of the penem, carbapenem prodrug, penicillin, penicillin sulfone, clavam, cephem, and monobactam β-lactam sub-families (Fig. S19–S21, ESI†). In some cases, *e.g.* clavulanate (Fig. S19H, ESI†) and moxalactam (Fig. S21F, ESI†), (partial) inhibitor fragmentation was observed. There was no clear correlation between a propensity to react covalently and M^pro^ inhibition; in some cases evidence for partial covalent modification, but no inhibition was observed (Fig. S17, ESI†). Covalent modification was observed with **1**, but only to a small (<10%) extent with **2**, suggesting the latter likely inhibits principally by a non-covalent interaction (Fig. 3C–H). After Cys-145 blocking with N3, no reaction with **1** was observed (Fig. 3G). Minor further modification of Cys-145 reacted M^pro^ was observed with **2** (Fig. 3H), suggesting the low levels of covalent modification by **2** do not solely involve Cys-145.

In summary, SPE-MS is a useful method for M^pro^ assays enabling analysis of inhibition by both substrate depletion/product formation. The method complements reported *in vitro* M^pro^ assays and compares favourably to those in terms of its robustness and ability to enable efficient high-throughput screening/repurposing efforts. The SPE-MS assay also enables ready analysis of covalent M^pro^ modification and use of M^pro^ reacted with a selective inhibitor such as N3/TPCK informs on whether covalent reaction of a test inhibitor occur at the active site or not.

Although the available evidence implies that β-lactams can inhibit M^pro^ non-covalently, the observation that some react with and inhibit M^pro^ by covalent active site modification should promote interest in the development of inhibitors for M^pro^ and other thiol proteases working *via* cysteinyl *S*-acylation. By contrast with *S*-alkylating inhibitors, which can have toxicity issues, *S*-acylation has not been widely explored for nucleophilic cysteine proteases.

The identification of β-lactam containing M^pro^ inhibitors with structures closely related to drugs should promote work on the development of related compounds for progression towards clinical use for treatment of COVID-19 and viral diseases.

We thank the Oxford COVID fund and its generous donors, the BBSRC, MRC, the Wellcome Trust, Cancer Research UK and GSK for funding, and D. Ebner (TDI, Oxford) for supplying the compound libraries. We thank present and past group members including T. Suits and J. Brem and support staff including A. Hardy, Diamond and RCaH support staff who have supported our COVID-19 work, including those who made compounds for initial screening. T. R. M. was supported by the BBSRC (BB/M011224/1). T. J. thanks the EPSRC Oxford-GSK-Crick Program (EP/R512060/1) and GSK. H. T. H. C. thanks the EPSRC SBM CDT (EP/L015838/1) and the Clarendon Fund. We thank J. E. Baldwin, R. M. Adlington and R.D.G. Cooper for encouragement and inspiration.

## Conflicts of interest

There are no conflicts to declare.

## Supplementary Material

Supplementary information
