# Supplementary material for: Mass spectrometry reveals potential of β-lactams as SARS-CoV-2 Mpro inhibitors
Source: Chem Commun (Camb). 2021 Jan 19;57(12):1430–3. doi: 10.1039/d0cc06870e (PMC8006714; doi:10.1039/d0cc06870e)
Supplement: Supplementary file 1 [file CC-057-D0CC06870E-s001.pdf]

Electronic Supplementary Material (ESI) for ChemComm.
This journal is © The Royal Society of Chemistry 2020

Supplementary Information

Mass spectrometry reveals potential of β-lactams as SARS-CoV-2 M^{pro} inhibitors

Tika R. Malla,[a] Anthony Tumber,[a] Tobias John,[a] Lennart Brewitz,[a] Claire Strain-Damerell,[b,c] C. David Owen,[b,c] Petra Lukacik,[b,c] H.T. Henry Chan,[a] Pratheesh Maheswaran,[a] Eidarus Salah,[a] Fernanda Duarte,[a] Haitao Yang,[d] Zihe Rao,[d] Martin A. Walsh[b,c] and Christopher J. Schofield[a*]

[a.] *Chemistry Research Laboratory, Department of Chemistry, 12 Mansfield Road, Oxford, OX1 3TA, United Kingdom.* E-mail: christopher.schofield@chem.ox.ac.uk

[b.] *Diamond Light Source, Harwell Science & Innovation Campus, Didcot, Oxfordshire OX11 0DE, United Kingdom.*

[c.] *Research Complex at Harwell, Harwell Science & Innovation Campus, Didcot, Oxfordshire OX11 0FA, United Kingdom.*

[d.] *Shanghai Institute for Advanced Immunochemical Studies and School of Life Science and Technology, ShanghaiTech University, Shanghai, China.*

Contents

Experimental section. 2
Protein production and purification 3
Peptide synthesis and purification 3
Inhibitor synthesis 3
General information on the M^{pro} mass spectrometry assays 4
Kinetic parameters of M^{pro} 4
Single concentration M^{pro} inhibition assays 4
IC_{50} value determinations 4
Protein Observed Mass Spectrometry Assay 4
Figure S1. Steady state kinetics for M^{pro} with the 11mer TSAVLQ/SGFRK-NH_2 substrate 6
Figure S2. Steady state kinetics for M^{pro} with the 37mer substrate 7
Table S1. Comparison of the M^{pro} kinetic parameters obtained using SPE-MS with those reported. 8
Figure S3. Dose response curves of reported M^{pro} inhibitors determined using SPE-MS. 9
Figure S4. N3 binds M^{pro} through covalent modification. 10
Table S2. RMSD of cysteines in N3 bound M^{pro} (PDB ID: 6LU7) and apo-M^{pro} (PDB ID: 6YB7) 10
Figure S5. The cysteine residues in M^{pro} 11
Figure S6. Ebselen covalently reacts with M^{pro} as observed by SPE-MS 12
Figure S7. M^{pro} SPE-MS assays with drugs 13
Table S3. FDA-approved small molecules for therapeutic use in humans which inhibit >80% M^{pro} activity at a fixed concentration as observed by the SPE-MS assay. 14
Table S4. Small molecules from LOPAC^{1280} library which inhibit ≥80% M^{pro} activity at a fixed concentration as observed by the SPE-MS assay 15
Figure S8. Dose response curves for selected M^{pro} inhibitors identified from the library screens. 17
Figure S9. IPA-3 reacts with M^{pro} 18
Figure S10. NSC 95397 reacts with M^{pro} 19
Figure S11. BAY 11-7082 reacts with M^{pro} 20
Figure S12. N_α-p-Toluenesulfonyl-L-lysine chloromethyl ketone (TLCK) reacts with M^{pro}. 21
Figure S13. N-p-Toluenesulfonyl-L-phenylalanine chloromethyl ketone (TPCK) reacts with M^{pro} 22
Figure S14. Bismuth(III) subsalicylate does not covalently react with M^{pro} to give a stable acyl-enzyme complex. 23
Figure S15. 5-Thioguanine does not covalently react with M^{pro} to give a stable acyl-enzyme complex 24
Table S5. Several β-lactam M^{pro} inhibitors identified from library screen (Figure S7). 25
Figure S16. Zn ions inhibit M^{pro} 26
Figure S17. Assaying inhibition of M^{pro} by β-lactam antibiotics. 27
Figure S18. Docking poses of β-lactams at the M^{pro} active site based on a reported crystal structure (PDB: 6LU7) 28
Figure S19. Some β-lactams react with M^{pro}. 29
Figure S20. Several penicillins react with M^{pro} 30
Figure S21. Several cephalosporins react with M^{pro} 32
References 33

Experimental section:

Protein production and purification

The reported pGFX-6p1-M^{pro} plasmid[1] was used for M^{pro} protein production.[2] In brief, the pGFX-6p1-M^{pro} plasmid was transformed into a competent *Escherichia coli* expression cell line based on the BL21(DE3)-R3-pRARE. Multiple colonies were used to inoculate an LB media starter culture supplied with 100 µg/mL carbenicillin. The starter culture was incubated (37°C, 200 rpm) until an OD600 ≤ 2 was reached (~8 h). 1% (v/v) of the starter culture was used to inoculate pre-warmed autoinduction medium (Formedium, Terrific Broth base including trace elements) supplemented with 1% (v/v) glycerol and 50 µg/mL carbenicillin. Cells were grown at 37°C (200 rpm) for 5 h and at 18°C overnight (200 rpm). Cells were harvested by centrifugation (10 krpm, 30 min, 4°C).

The resulting cell pellet was suspended in lysis buffer (50 mM Tris, pH 8, 300 mM NaCl, 10 mM imidazole, 0.03 µg/ml benzonase) at a ratio of 1:3 (w/v). Cells were lysed using an Emulsiflex high-pressure homogeniser (Avestin Inc) (3 passes, 30 kpsi, 4°C). The lysate was centrifuged (50000 g, 30 min-60 min, 4°C) and filtered (0.22 µm). The filtrate was incubated with 10ml 50% (v/v) pre-equilibrated His60 Ni Superflow resin (Clontech Laboratories) (with agitation, 1 h, 4°C) and then applied to a gravity flow column. The column was washed (50 mM Tris pH 8, 300 mM NaCl, 25 mM imidazole), and the M^{pro} protein was eluted with elution buffer (50 mM Tris, pH 8, 300 mM NaCl, 500 mM imidazole). To remove the M^{pro} poly-histidine tag, the N-terminal His tagged HRV 3C protease was added to the eluted M^{pro} fractions (1:10 (w/w) protease:M^{pro}). The mixture was dialysed overnight into 50 mM Tris pH 8, 300 mM NaCl, 0.5 mM TCEP at 4 °C. Mpro was purified by reverse His purification using a 5 mL HisTrap FF column (GE Healthcare) eluting with wash buffer (50 mM Tris pH 8, 300 mM NaCl, 25 mM imidazole). Purified M^{pro} fractions were pooled and concentrated (< 5mL) using an Amicon® Ultra 10 MWCO centrifugal filter unit (Merck Millipore). The concentrate was loaded onto a S200 pg 16/60 size exclusion column (GE Healthcare), eluting at a flow rate of 1 mL/min (50 mM Tris pH 8, 300 mM NaCl). M^{pro} fractions were concentrated to 36 mg/mL using a 10 kDa MWCO centrifugal filter. M^{pro} was >95% pure by SDS PAGE analysis and had the anticipated mass of 33786 ± 1 Da (calculated: 33796.64 Da).

Peptide synthesis and purification

The 11-mer M^{pro} substrate peptide TSAVLQ/SGFRK-NH_2, corresponding to the N-terminal self-cleavage site of M^{pro} on pp1a/b, was synthesised as a C-terminal amide by microwave-assisted solid-phase peptide synthesis (SPPS) as reported.[3] In brief, the M^{pro} substrate peptide was synthesized on a 0.1 mmol scale from the C- to N-terminus on Rink amide-MBHA resin (100–200 mesh, 0.6–0.8 mmol. g^{-1} loading, AGTC Bioproducts) using a LibertyBlue peptide synthesizer (CEM) and *N*-Fmoc protected α-amino acids (CS Bio, Novabiochem, Sigma-Aldrich, TCI, Alfa Aesar, Merck or AGTC Bioproducts). A mixture of *N,N′*-diisopropylcarbodiimide (TCI Europe) and Oxyma Pure (Merck) in DMF was used for coupling and 20% (v/v) piperidine in DMF (peptide synthesis grade, AGTC Bioproducts) for Fmoc deprotection. Iterative cycles of peptide coupling and deprotection were performed using the manufacturer's protocol. After the final Fmoc-deprotection step, the resin was washed with CH_2Cl_2, dried in air, then treated with 5 mL of the deprotection solution (2.5%:2.5%:2.5%:92.5%) (v/v) of 1,3-dimethoxybenzene, triisopropylsilane, MilliQ water and trifluoroacetic acid) (4 h at ambient temperature). The resulting mixture was filtered; the filtrate was diluted with cold Et_2O (45 mL) to precipitate the peptide. The resulting suspension was centrifuged (4255 g, 10 min, 4.0 °C) and the supernatant decanted. The solid peptide was dried in air, dissolved in MilliQ water, then lyophilised. The peptide was purified by reverse phase HPLC (20 mL/min; linear gradient over 10 min: 2%→20% acetonitrile in water, each containing 0.1% (v/v) formic acid; t_R = 8 min) using a Shimadzu HPLC purification system (composed of DGU-20A, 2 LC-20AR, CBM-20A, SPD-20A, and FRC-10A units) equipped with a NX-C18 LC column (250 × 21.2 mm, 110 Å; Phenomenex Gemini).

The purified peptide was dissolved in DMSO and its concentration was determined by ^1H NMR using an AVIII 700 machine (Bruker) equipped with a 5 mm $^1H(^{13}C/^{15}N)$ inverse cryoprobe: 16 µL of the DMSO peptide solution were added to 143 µL DMSO-d_6 and 1 µL of 1 mg/mL 3-(trimethylsilyl)propionic-2,2,3,3-d_4 acid sodium salt (TSP; Apollo Scientific) (Acquisition parameters: d1 = 30 s, ns = 256). Peaks were assigned in MestreNova using the global spectrum deconvolution algorithm. The peptide concentration was then calculated using the following equation[4]:

$$\frac{Mx}{My} = \frac{Ix}{Iy} \times \frac{Ny}{Nx},$$

where,

$\frac{Mx}{My}$ is the molar ratio of TSP and the 11-mer substrate peptide respectively $\frac{Ix}{Iy}$ is the ratio of signal intensity corresponding to the methyl protons of TSP and the 11-mer substrate peptide (averaged for the Ala, Val, and Leu residues) respectively, and $\frac{Ny}{Nx}$ is the number of nuclei responsible for the signal intensity for 11-mer substrate (averaged for the Ala, Val, and Leu residues) and TSP respectively.

Peptide aliquots were prepared and stored at -20°C.

Inhibitor synthesis

Syntheses of the M^{pro} inhibitors N3,[1] **1,**[5] **2,**[6] **3,**[7] **4,**[8] and **5**[9] have been reported.

General information on the M^{pro} mass spectrometry assays

M^{pro} MS assays were performed using freshly thawed M^{pro}; thawing was not repeated because refreezing and thawing M^{pro} reduces M^{pro} activity, so affecting assay robustness and reproducibility. Freshly thawed M^{pro} was diluted to 15 µM in freshly prepared 20 mM HEPES (Gibco) buffer pH 7.5, 300 mM NaCl for all MS assays with the exception of protein observe MS assays, for which M^{pro} was diluted to 1 µM in freshly prepared reaction buffer (20 mM HEPES pH 7.5, 50 mM NaCl). LCMS (Merck, Supelco) grade solvents were used for MS analyses and to prepare buffers. All assays were performed in 384 well polypropylene plates (Greiner) in the reaction buffer. All compounds were purchased from Sigma Aldrich, Apollo and Fluorochem. All compounds were dry dispensed using an Echo 550 acoustic dispenser (LabCyte) unless otherwise stated. The substrate peptide and the enzyme was diluted in the reaction buffer to prepare stock solutions, 2 fold concentrated relative to the final concentration in the assay. M^{pro}, peptide substrate, and formic acid solutions were all dispensed across 384 well plates using a Multidrop Combi dispenser (Thermo Scientific), the plates were then centrifuged (500 x g, 15s, Axygen Plate Spinner Centrifuge, Corning).

M^{pro} activity was assayed using a RapidFire (RF) 365 high-throughput sampling robot (Agilent) connected to an iFunnel Agilent 6550 accurate mass quadrupole time-of-flight (Q-TOF) mass spectrometer. The spectrometer was operated in the positive ion ionisation mode with following operating parameters for all assays: capillary voltage (4000 V), nozzle voltage (1000 V), fragmentor voltage (365 V), drying gas temperature (280 °C), gas flow (13 L/min), sheath gas temperature (350 °C), sheath gas flow (12 L/min); except for protein observe MS assays, for which the gas temperature was reduced to 225 °C. The peptide/protein sample was loaded onto a solid-phase extraction (SPE) C4-cartridge, which was then washed with 0.1% (v/v) aqueous formic acid to remove non-volatile buffer salts (5.5 s, 1.5 mL/min) and with aqueous 85% (v/v) acetonitrile containing 0.1% (v/v) formic acid (5.5 s, 1.25 mL/min) to elute the peptides/protein. The cartridge was re-equilibrated with 0.1% (v/v) aqueous formic acid (0.5 s, 1.25 mL/min). Each sample aspiration step was followed by an aqueous wash before the next protein sample was injected onto the SPE cartridge.

Kinetic parameters of M^{pro}

The kinetic parameters of M^{pro} were determined by monitoring M^{pro}-catalysed cleavage of TSAVLQ/SGFRK-NH_2 over time using 16 different peptide concentrations (the final DMSO concentration was >1% (v/v) in each experiment, **Figure S1**). The peptide was diluted (1:100) with the reaction buffer (20 mM HEPES, pH 7.5, 50 mM NaCl) to 0.5 mL reaction volume. Initially, a no-enzyme control was analysed using SPE-MS before M^{pro} was added to the mixture (0.15 µM final concentration, based on the M^{pro} monomer mass) using SPE-MS.

RapidFire integrator software (Agilent) was used to extract and integrate the m/z (+1) charge states of both the substrate peptide (1191.67 Da) and the N-terminal product peptide (TSAVLQ; 617.34 Da). The basic C-terminal cleavage product (SGFRK-$NH_2$was not detected, most likely due to poor retention on SPE cartridge under these conditions and hence was not included in subsequent calculations. The percentage conversion (product peak integral/(product peak integral + substrate peak integral)*100) was calculated in Microsoft Excel. The slopes of the initial reaction rate was fitted with the Michaelis-Menten equation (E_t= 0.15 µM) using non-linear regression (GraphPad Prism 8) to obtain the K_m and k_{cat}-values (**Figure S1**). The experiments were performed in independent duplicates (n = 2; mean ± standard deviation, SD).

Single concentration M^{pro} inhibition assays

The final compound assay concentrations were 20 µM, 50 µM, or 100 µM; DMSO and ebselen were used as negative and positive inhibition controls, respectively. M^{pro} (0.30 µM) was dispensed across the plate (25 µL/well); the resulting mixture was incubated for 30 or 60 minutes at ambient temperature. TSAVLQ/SGFRK-NH_2 peptide (4 µM) was dispensed to the mixture (25 µL/well), which was incubated (10 minutes), then quenched by addition of 10% (v/v) aqueous formic acid (5 µL/well). The individual reactions were analysed using SPE-MS. Data were extracted and processed as described for the kinetic M^{pro} assays above and the normalized percentage inhibition was determined in Microsoft Excel with respect to the positive and negative inhibition controls. Errors values were determined with respect to technical duplicates (n = 2; mean ± SD).

IC_{50} value determinations

M^{pro} inhibitors were dry dispensed in an 11 point 3 fold dilution series (100 µM top concentration). Ebselen and DMSO were used as positive and negative inhibition controls, respectively. M^{pro} (0.30 µM) was dispensed across the plate (25 µL/well) and the resulting mixture was incubated (30 or 60 minutes) at ambient temperature. TSAVLQ/SGFRK-NH_2 peptide solution (4 µM) was then dispensed to the mixture (25 µL/well). Reactions were incubated (10 minutes), then quenched by addition of 10% (v/v) aqueous formic acid (5 µL/well). Data were extracted and processed as described for the kinetic M^{pro} assays above. IC_{50} curves were generated using non-linear regression and normalized with respect to the positive and negative inhibition controls (GraphPad Prism 8). IC_{50}-values are reported as the mean of two independently determined IC_{50}-curves each composed of technical duplicates (n = 2; mean ± SD). Signal to noise (S/N) and Z'-factor were calculated in Microsoft Excel for each plate analysed.[8]

Protein Observed Mass Spectrometry Assay

For the initial screening of β-lactams, β-lactam solutions (10 mM in DMSO) were transferred in quadruplicate from a 96 well plate to a 384 well plate (1 µL/well) using a CyBio Liquid Handler (Analytik Jena AG). M^{pro} (1.0 µM) in the reaction was dispensed across the plate (100 µL/well) using a Multidrop Combi dispenser (Thermo Scientific) and the resulting mixture was incubated for a minimum of 1 h, 5 h, 19 h, and 24 h at ambient temperature before analysis using SPE-MS.

For selected M^{pro} inhibitors identified from the initial screening of β-lactams or from the screening of small-molecule libraries (LOPAC^{1280} (Sigma Aldrich) or the small-molecule library containing FDA approved compounds (Pharmacon) for human use), inhibitor solutions (20 mM in DMSO) were diluted to 1 mM with the reaction buffer and transferred to a 384 well plate (2 µL/well). M^{pro} (1.0 µM) was dispensed across the plate (100 µL/well) using a Multidrop Combi dispenser (Thermo Scientific) and the resulting mixture was incubated for a minimum of (5 min and 1 h) or (25 min, 1 h , 16 h and 24 h) at ambient temperature before analysis using SPE-MS.

To investigate whether the identified M^{pro} inhibitors selectively modify the M^{pro} active site Cys-145, M^{pro} (1 µM) was incubated with the reported Cys-145-selective M^{pro} inhibitor N3[1] (3 µM) on ice for 3 h in 7.5 mL reaction buffer (20 mM HEPES, pH 7.5, 50 mM NaCl). The inhibitor of interest (20 mM in DMSO) was diluted to 1 mM with the reaction buffer and transferred to a 384 well plate (2 µL/well). M^{pro}/N3 mixture was dispensed to the inhibitor of interest on a 384 well plate (100 µL/well) and the resulting mixture was incubated at ambient temperature for 5 min and 16 h, respectively, before analysis using SPE-MS.

Protein observed mass spectra were acquired (at least) in independent duplicates using SPE-MS with the instrument parameters as stated above. Only one representative of the data are shown as all duplicates were apparently identical. The reactions were not quenched, thus, each subsequent sample was injected with a delay from the primary injection time (stated in the individual figures). Protein spectra were deconvoluted (m/z range: 10-60 kDa, m/z range 850-1350 Da, mass step: 1 Da) using the MaxEnt1 function in Agilent MassHunter Version 7. The deconvoluted files were extracted, normalised and plotted (GraphPad Prism 8).

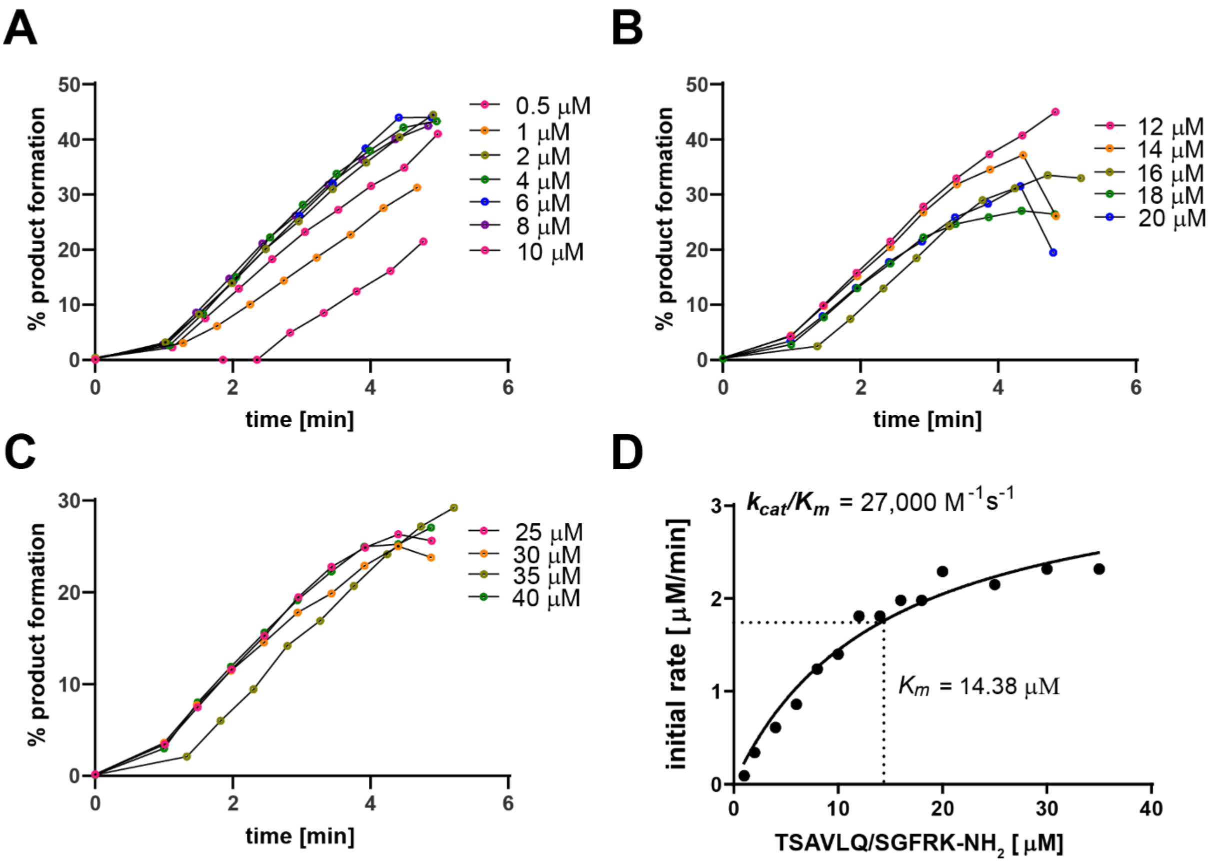

Figure S1. Steady state kinetic analyses for M^{pro} with the 11mer TSAVLQ/SGFRK-NH_2 substrate sequence. M^{pro} (0.15 µM) was incubated with different concentrations of the 11-mer peptide substrate (**A**) 0.5-10 µM, (**B**) 12-20 µM and (**C**) 25-40 µM with the conversion monitored by SPE-MS. (**D**) Plot of initial rate versus all substrate concentrations fitted using non-linear regression. The Michaelis-Menten equation gave k_{cat} = 23.47 min^{-1} and K_m = 14.38 µM. Note that interconversion between monomeric and dimeric forms of M^{pro} may cause complexity in its kinetics. Refer to Experimental Section for assay details.

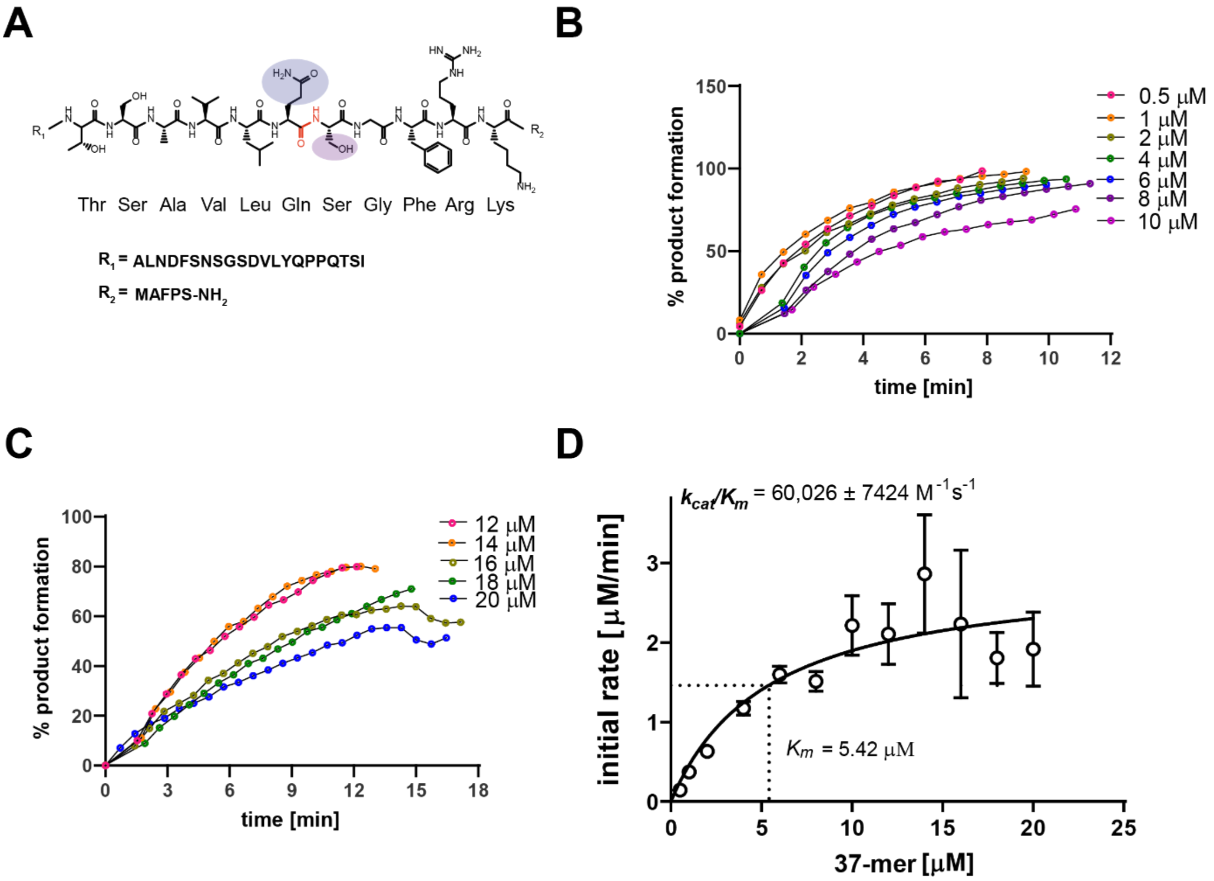

Figure S2. Steady state kinetics for M^{pro} with the 37mer substrate. (**A**) The 37-mer substrate with analogous residues of the 11-mer substrate in 'bond form' (Figure S1. The substrate (Gln/blue) P1 position and P1' (Ser/purple) residues are shaded. M^{pro} (0.15 µM) was incubated with different concentrations of the 37-mer peptide substrate: (**B**) 0.5-10 µM and (**C**) 12-20 µM with the conversion monitored by SPE-MS. (**D**) Plot of initial rate versus all substrate concentrations fitted using non-linear regression. The Michaelis-Menten equation was used to determine kinetic parameters: k_{cat} = 19.5 min^{-1} and K_{m} = 5.42 µM. Each data point represents the mean of two independent repeats (n=2 ± standard deviation (SD)). Note that interconversion between monomeric and dimeric forms of M^{pro} may cause complexity in its kinetics. Refer to Experimental Section for assay details.

Table S1. Comparison of the M^{pro} kinetic parameters obtained using SPE-MS with those reported. The kinetic parameters of SARS-CoV-2 M^{pro} determined using SPE-MS (Entries 12 and 13) are in the range of those recently reported for SARS-CoV-2 M^{pro}. Note that the catalytic efficiency of SARS-CoV-2 Mpro is slightly higher than that for SARS-CoV, possibly due to the apparent close packing of M^{pro} domain III in SARS-CoV-2 M^{pro}.[10] SARS-CoV and SARS-CoV-2 M^{pro} share 98% sequence identity.[11] Note that the N-terminal residue (Ser-1) has been shown to be important for M^{pro} dimerization.[2]

		Substrate	k_{cat}/k_m ($M^{-1}s^{-1}$)	Method
1	SARS-CoV	^{a}**Abz**-SVTLQ/SG-Tyr(NO_2)R	20	FRET[12]
2	SARS-CoV	TSAVLQ/SGFRK-NH_2	176 ± 10	RP-HPLC[13]
3	SARS-CoV	^{b}**Dabcyl**-LAQ/AVRSSSR-**Edans**	3666	FRET[14]
4	SARS-CoV	^{c}**Mca**-AVLQ/SGFR-Lys(**Dnp**)-Lys-NH_2	26500	FRET[2]
5	SARS-CoV	^{b}**Dabcyl**-SAVLQ/SGFRK-**Edans**/ TSAVLQ/SGFRKW	29000/2400	FRET/RP-HPLC[15]
6	SARS-CoV	^{a}**Abz**-SVTLQSG-Y(NO_2)-R	10000 ± 3333	FRET[16]
7	SARS-CoV	(ARLG-NH_2)-Rhodamine	50	FRET[17]
8	SARS-CoV	^{b}**Dabcyl**-KTSAVLQ/SGFRKM-E(**Edans**)	3011.3 ± 294.6	FRET [10]
9	SARS-CoV-2	^{b}**Dabcyl**-KTSAVLQ/SGFRKM-E(**Edans**)	3426.1 ± 416.9	FRET[10]
10	SARS-CoV-2	^{a}**Abz**-SVTLQSG-Y(NO_2)-R	30000 ± 6667	FRET[16]
11	SARS-CoV-2	^{c}**Mca**–AVLQ/SGFRK(**Dnp**)K	28500	FRET [1]
12	SARS-CoV-2	TSAVLQ/SGFRK-NH_2	27000	SPE-MS
13	SARS-CoV-2	ALNDFSNSGSDVLYQPPQTSITSAVLQ/SG FRKMAFPS-NH_2	60026 ± 7424	SPE-MS

$^{a)}$ **Abz**: 2-aminobenzoyl.

$^{b)}$ **Dabcyl**: 4-([4-(dimethylamino)phenyl]-azo)-benzoic acid; **Edans**: 5-((2-aminoethyl)amino)naphthalene-1-sulfonic acid.

$^{c)}$ **Mca**: 7-methoxycoumarin-4-acetic acid; **Dnp**: 2,4-dinitrophenyl.

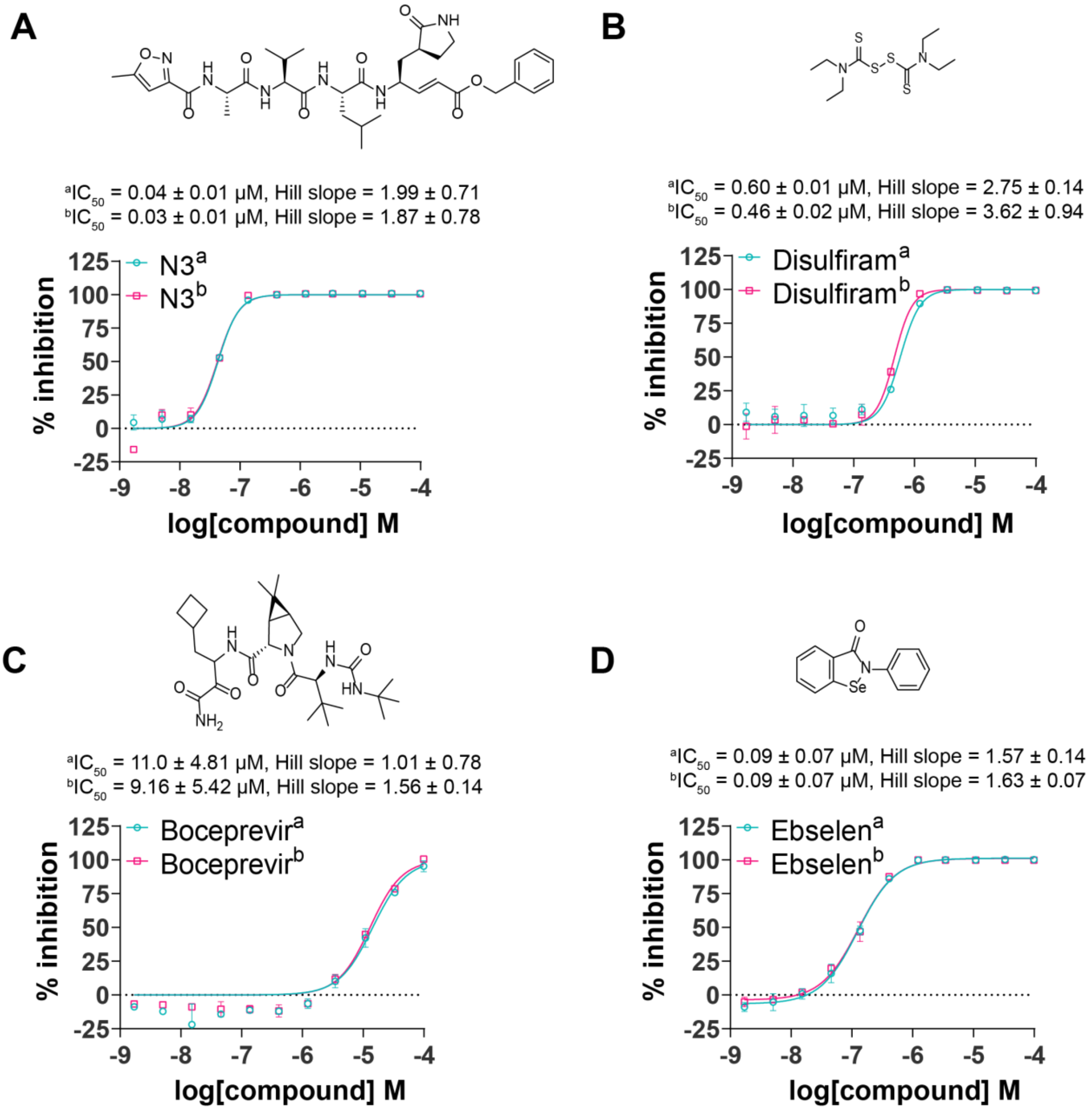

Figure S3. Dose response curves of reported M^{pro} inhibitors determined using SPE-MS. (A) N3[1], (B) disulfiram, (C) boceprevir, (D) ebselen. Each data point represents technical duplicates. IC_{50}s were calculated from two independent repeats each composed of technical duplicates (n=2 ± SD). Conditions: 0.15 µM M^{pro} and 2.0 µM TSAVLQSGFRK-NH_2 substrate peptide in the reaction buffer (20 mM HEPES, pH 7.5, 50 mM NaCl) with ([a]) 30 min compound preincubation (open square, turquoise) and ([b]) 60 min preincubation (open circle, magenta). Structures of the inhibitors are shown.

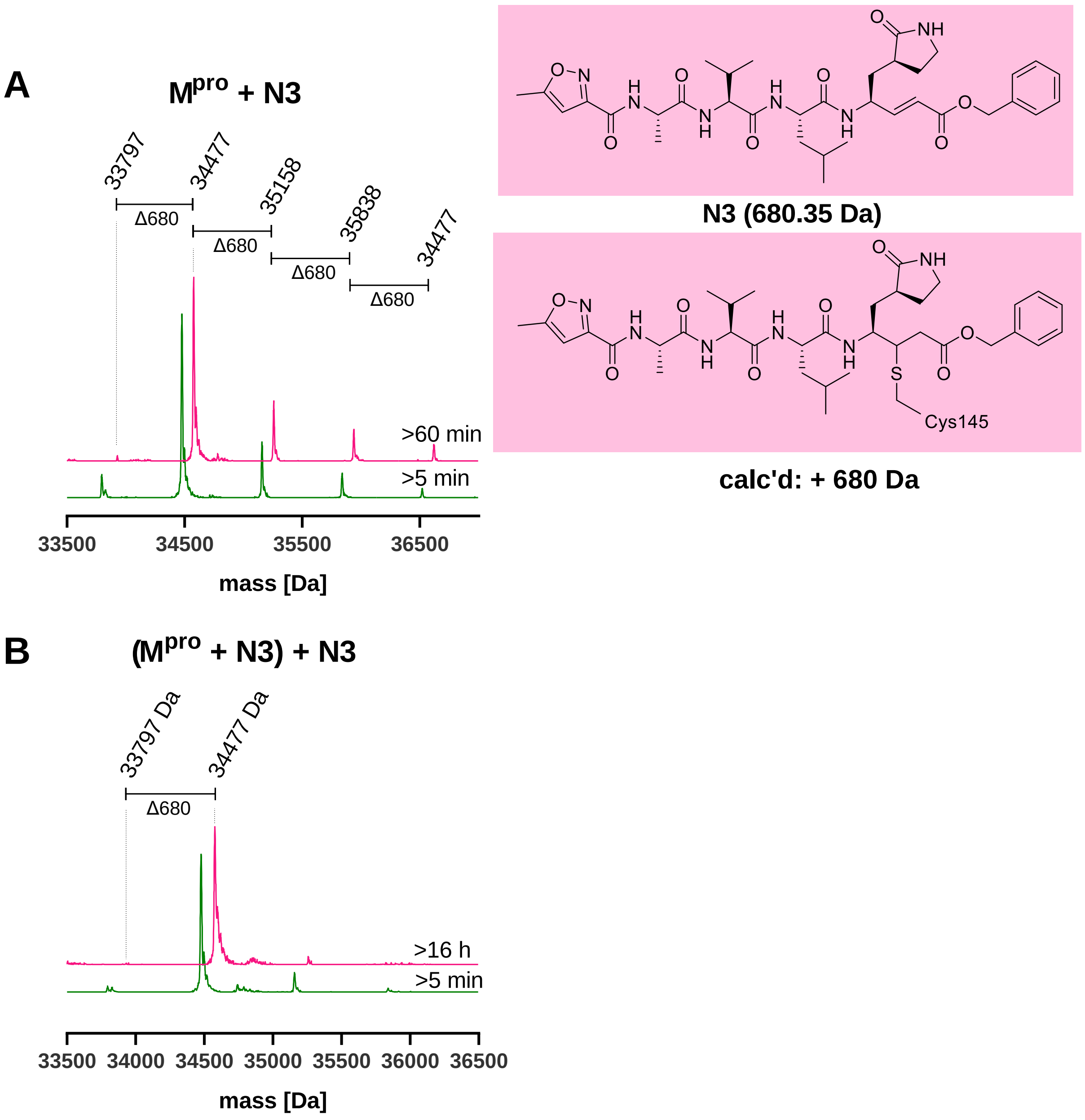

Figure S4. N3 binds M^{pro} through covalent modification. SPE-MS binding assays were performed as described in the Experimental Section using M^{pro} (1 µM) and N3[1] (3 or 20 µM). (A) Reaction with excess N3 (1:20, protein:inhibitor) results in more than one reaction with the M^{pro} monomer. (B) Reaction of a preincubated protein:N3 mixture (1:3 ratio) with excess N3 (20 µM) results in one major binding event, likely at the M^{pro} active site Cys-145 as independently shown by crystallographic analysis.[1] The black spectrum is control M^{pro} (33796 ± 1 Da) without an inhibitor. See Experimental Section for assay details. The structure of the inhibitor and the outcome of reaction with a nucleophilic cysteine are shown.

Table S2. RMSD comparison of cysteine conformations in N3 bound M^pro^ (PDB ID: 6LU7) and apo-M^pro^ (PDB ID: 6YB7). RMSD values were calculated for all cysteine residues of the N3 bound M^pro^ and the apo-M^pro^ structures using least square fitting of all cysteine atoms in COOT. The RMSDs observed for Cys-22, Cys-44, Cys-117 and Cys-156 are in the range of active site Cys-145 (magenta) RMSD. Two conformations for Cys-117 (occupancy = 0.6) and Cys-128 (occupancy = 0.8) are present in the apo-M^pro^ crystal structure; the RMSDs at the low occupancy conformations are in parentheses.

Residue	Cys-16	Cys-22	Cys-38	Cys-44	Cys-85	Cys-117	Cys-128	Cys-145	Cys-156	Cys-160	Cys-265	Cys-300
**RMSD**	0.08	0.13	0.04	0.14	0.04	0.13 (0.70)	0.79 (0.15)	0.12	0.13	0.06	0.07	0.07

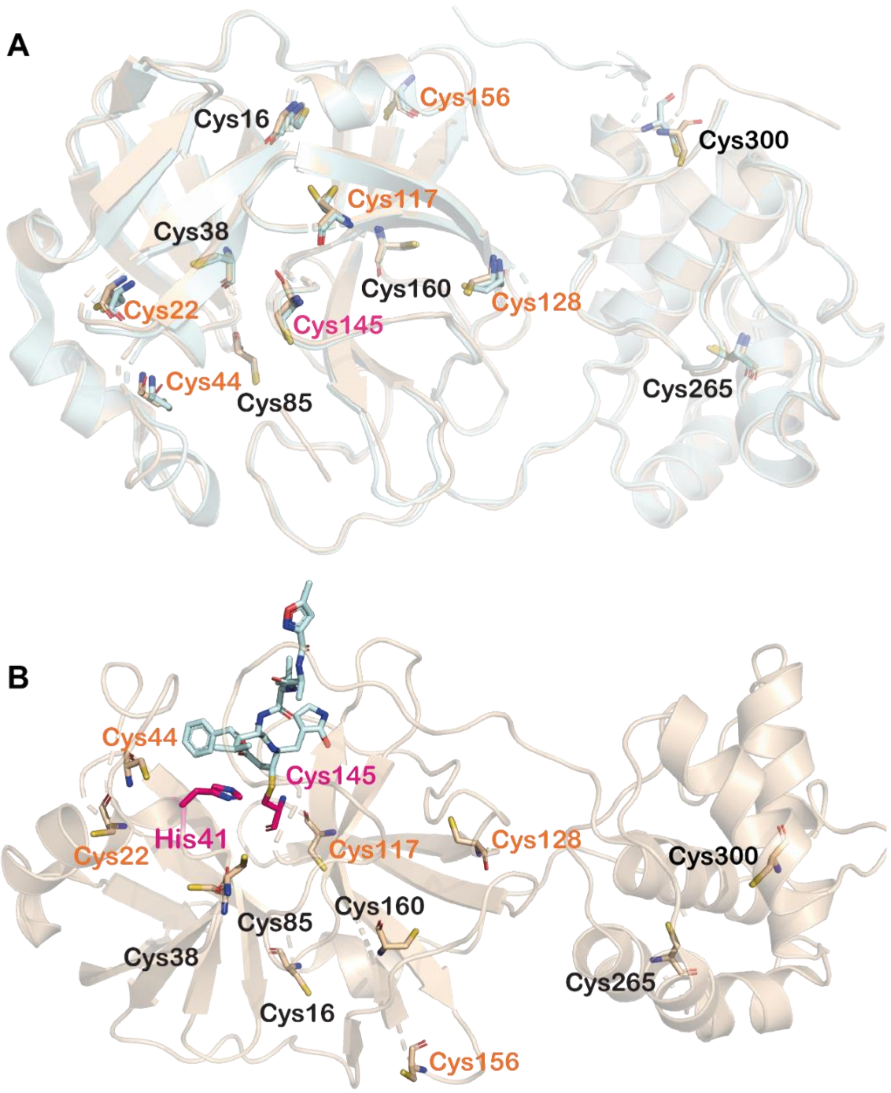

Figure S5. The cysteine residues in M^pro^. (**A**) Superimposition of an unreacted M^pro^ (**PDB ID: 6YB7**) and N3 bound M^pro^ (**PDB ID: 6LU7**).[1] The binding of N3 at M^pro^ does not trigger conformational changes in most M^pro^ cysteine residues as observed by crystallography. Cys-128 (RMSD = 0.79) experiences the largest conformational changes (highlighted in orange). Cys-22, Cys-44, Cys-117 and Cys-156 undergo comparable conformational change with the active site Cys-145. (**B**) Reaction of M^pro^ with N3 does not alter the conformations of other cysteines, nor apparently "shield" their thiols. RMSD values were calculated for specified residue using least square fitting for all atoms of the specified residue in COOT (Table S2).

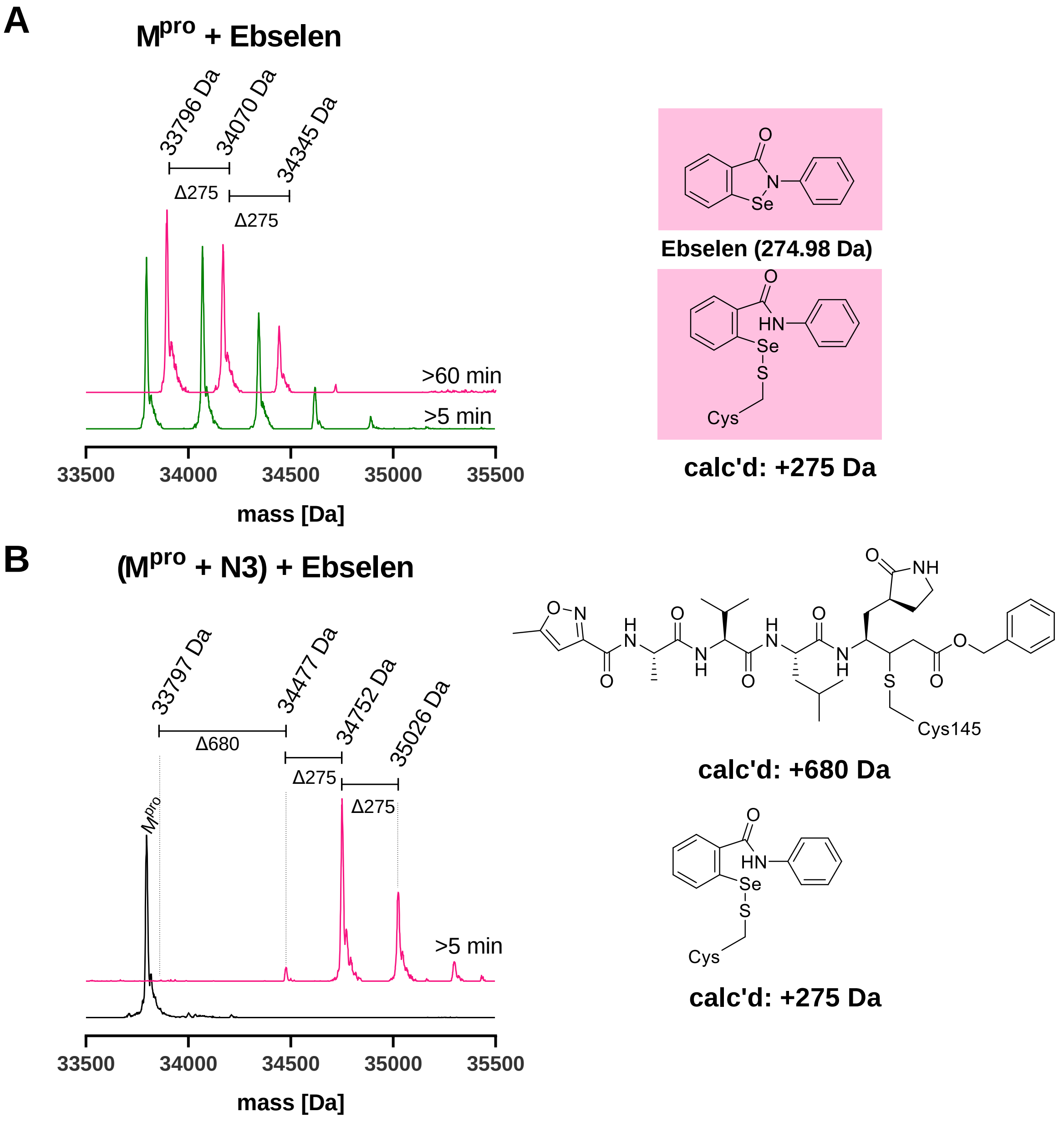

Figure S6. Ebselen covalently reacts multiple times with M^{pro} as observed by SPE-MS. (**A**) Ebselen covalently reacts with M^{pro} at multiple positions. Conditions: 1 µM M^{pro}, 20 µM ebselen in the reaction buffer (20 mM HEPES, pH 7.5, 50 mM NaCl). (**B**) Ebselen covalently modifies M^{pro} in the presence of N3. The results indicate that ebselen does not selectively react with Cys-145 of M^{pro}. Conditions: 1 µM M^{pro} preincubated with 3 µM N3, 20 µM ebselen in the reaction buffer (20 mM HEPES, pH 7.5, 50 mM NaCl). The black spectrum is for M^{pro} (33796 ± 1 Da) without inhibitor. Note ebselen may only be partially soluble in the assay buffer. See Experimental Section for details. The structure of the inhibitor and a proposed outcome of reaction with a nucleophilic cysteine are shown.

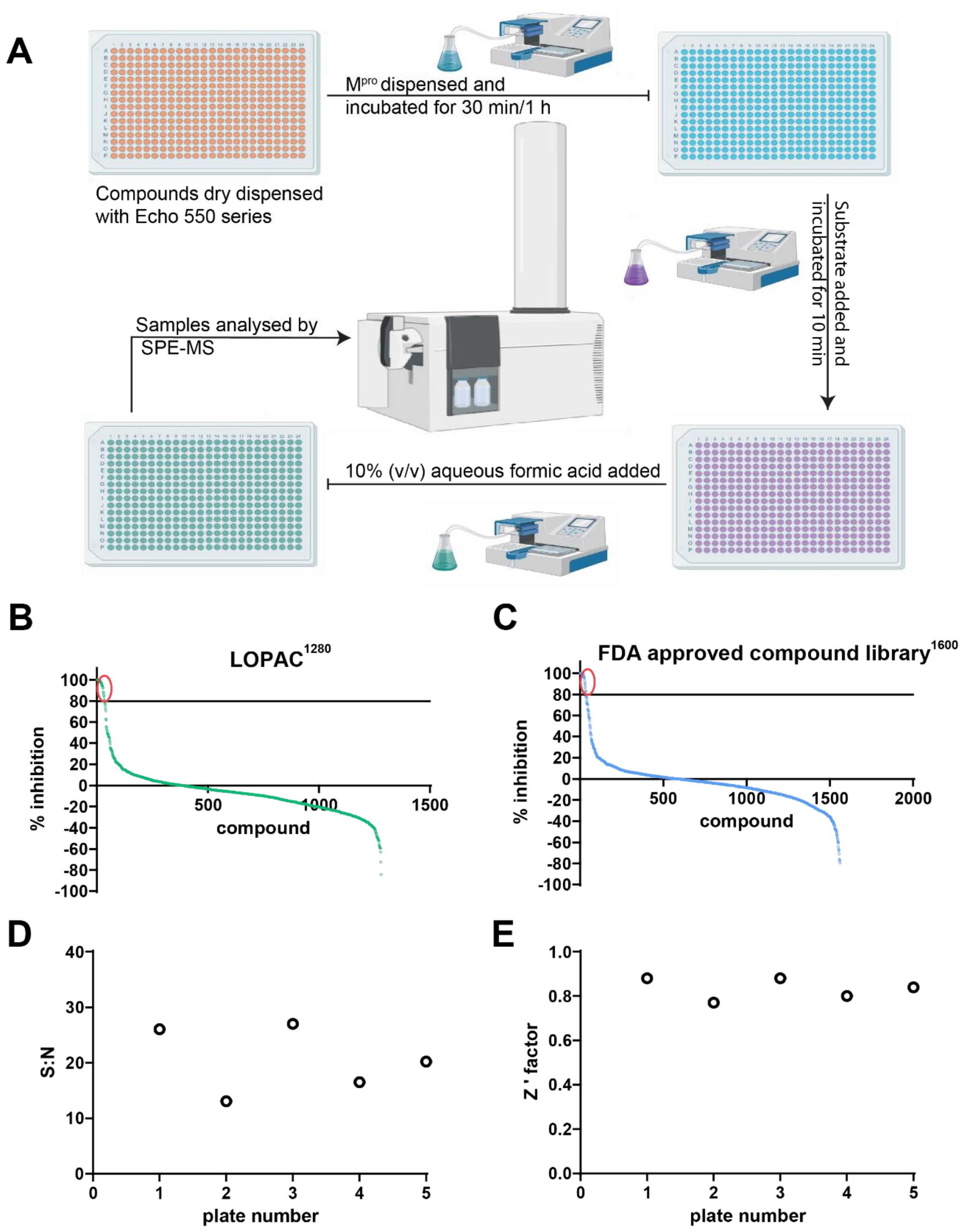

Figure S7. Mpro SPE-MS assays with drugs. (**A**)The experimental set up of SPE-MS based Mpro assay. Mpro was profiled against the (**B**) library of pharmacologically active compounds (LOPAC1280, Sigma-Aldrich), which contains 1280 bioactive small molecules and (**C**) library of small molecules approved by the FDA (1600 compounds, Pharmacon) for therapeutic use in humans (provided by the TDI, Oxford). As a measure of assay robustness both (**D**) signal/noise (S/N) and (**E**) the assay Z' factors were calculated for all 5 assay plates according to the literature.[18] Both values indicate that the assay is of high quality and robustness. Compounds that exhibited ≥80% inhibition in LOPAC1280 library and library of FDA approved small molecules are shown in **Table S2** and **Table S3**.

Table S3. FDA-approved small molecules for therapeutic use in humans which inhibit >80% M^{pro} activity at a fixed concentration as observed by the SPE-MS assay. Conditions: 20 µM compounds, 0.15 µM M^{pro}, 2.0 µM TSAVLQ/SGFRK-NH_2 in the reaction buffer (20 mM HEPES, pH 7.5, 50 mM NaCl). See Experimental Section for assay details.

**Compound**	**% M^{pro} inhibition at 20 µM**
Cisplatin	100.0
Sanguinarium chloride	100.0
Pipenzolate bromide	100.0
Oltipraz	99.6
Disulfiram[1]	99.1
Ebselen[1]	99.1
Apomorphine hydrochloride	97.0
Carmustine	96.0
Nitroxoline	96.0
Benzyl isothiocyanate	95.8
Colistin sulfate	95.8
Bismuth subsalicylate	92.9
Thioguanine	90.5
Netilmicin sulfate	89.1
Erythrosine sodium	86.5
Chlorophyllide copper complex sodium salt	86.5
Methazolamide	82.9

Table S4. Small molecules from LOPAC[1280] library which inhibit ≥80% M^{pro} activity at a fixed concentration as observed by the SPE-MS assay. Conditions: 20 µM compounds, 0.15 µM M^{pro}, 2.0 µM TSAVLQ/SGFRK-NH_2 in the reaction buffer (20 mM HEPES, pH 7.5, 50 mM NaCl). See Experimental Section for assay details.

**Compound**	**% M^{pro} inhibition at 20 µM**
Auranofin[19]	100.0
Sch-202676 hydrobromide	100.0
(*R*)-(−)-Propylnorapomorphine hydrochloride	100.0
Apomorphine hydrochloride hemihydrate	100.0
N-Carbobenzyloxy-L-phenylanlanyl chloromethyl ketone	100.0
Guanidinyl-naltrindole di-trifluoroacetate	99.6
(*R*)-(−)-2,10,11-Trihydroxyaporphine hybrobromide	99.6
cis-Diammine(pyridine)chloroplatinum(II) chloride	99.6
6-Nitroso-1,2-benzopyrone	99.5
Iodoacetamide	99.3
1,4-Benzoquinone	99.3
Cisplatin	99.2
1,1'-Disulfanediyldinaphthalen-2-ol (IPA-3)	99.1
NSC 95397	99.0
Sanguinarine chloride	98.7
Myricetin	98.5
(*R*)-(−)-2,10,11-Trihydroxy-n-propylnoraporphine hydrobromide	98.3
Nordihydroguaiaretic acid from *Larrea divaricata* (creosote bush)	97.8
Tetraethylthiuram disulphide (disulfiram)	97.5
ZM 39923 hydrochloride	96.9
Aurothioglucose	96.6
Ammonium pyrrolidinedithiocarbamate	95.1
Bay 11-7085	95.0
Capsazepine	94.5
N-p-Toluenesulfonyl-*L*-phenylalanine chloromethyl ketone (TPCK)	94.1
Piceatannol	93.8
Cephalosporin C zinc salt	92.3
Bay 11-7082	92.0
(±)-2-Amino-6,7-dihydroxy-1,2,3,4-tetrahydronaphthalene hydrobromide (6,7-ADTN hydrobromide)	88.3
N_α-p-Toluenesulfonyl-*L*-lysine chloromethyl ketone hydrochloride (TLCK)	88.3

Pyrocatechol	85.4
Chelerythrine chloride	85.2
Carmustine	83.0
3,4-Dichloroisocoumarin	80.4

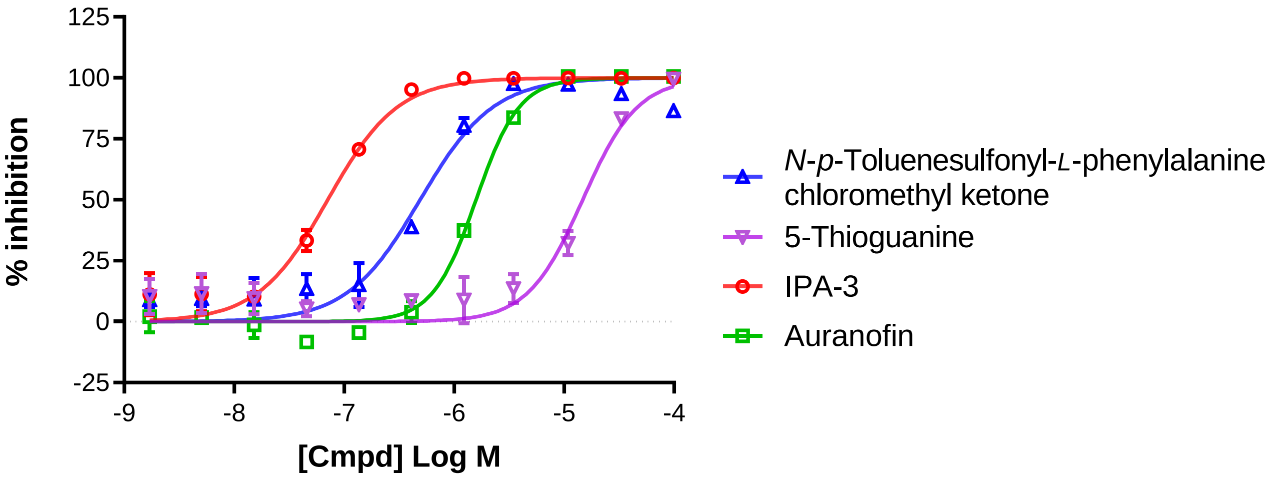

Figure S8. Dose response curves for selected M^{pro} inhibitors identified from the library screens. M^{pro} inhibition curves are shown for the selective p21 activated kinase (PAK) inhibitor IPA-3[20] (IC_{50} = 0.07 ± 0.03 µM, red circles), the anti-rheumatoid arthritis agent Auranofin[21] (IC_{50} = 1.47 ± 0.15 µM; green squares), the purine analogue 5-thioguanine (IC_{50} = 13.5 ± 1.71 µM; purple triangles), and the cysteine proteinase inhibitor N-p-toluenesulfonyl-L-phenylalanine chloromethyl ketone (TPCK)[22] (IC_{50} = 0.83 ± 0.47 µM; blue triangles). Each curve represents the mean of two technical duplicates. IC_{50} were calculated based on two independent repeats each composed of two technical duplicates (n=2, mean ± SD). The IC_{50} of IPA-3 was based on the mean of technical duplicates (mean ± SD). Conditions: 0.15 µM M^{pro}, 2.0 µM TSAVLQ/SGFRK-NH_2 substrate peptide in the reaction buffer (20 mM HEPES, pH 7.5, 50 mM NaCl, 20°C). See Experimental Section for assay details.

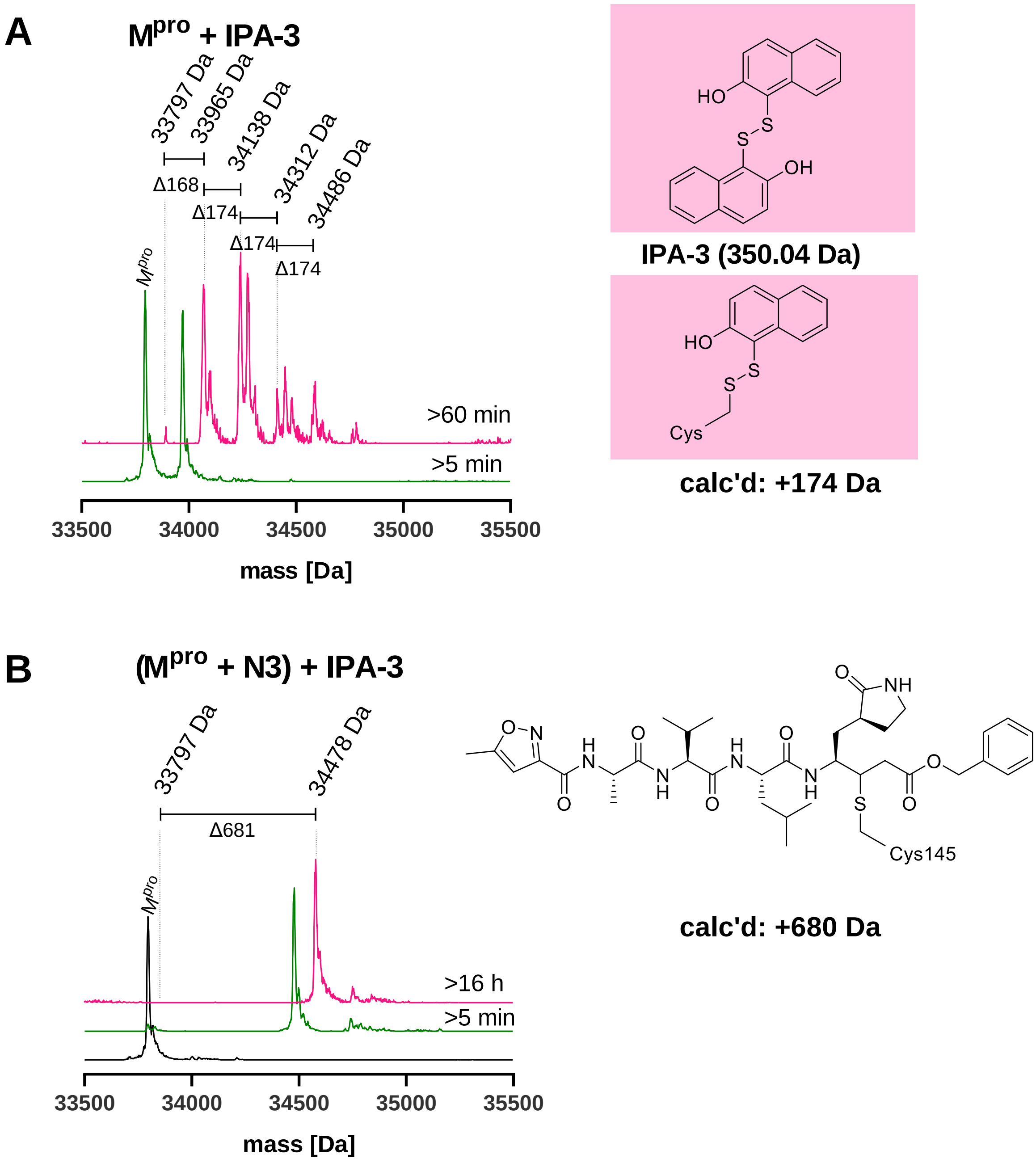

Figure S9. IPA-3 reacts with M^{pro}. (**A**) IPA-3 reacts with M^{pro} at multiple positions. Conditions: 1 µM M^{pro}, 20 µM IPA-3 in the reaction buffer (20 mM HEPES, pH 7.5, 50 mM NaCl). (**B**) IPA-3 does not (at least substantially) covalently modify M^{pro} in the presence of N3 inhibitor[1] suggesting that IPA-3 modifies the active site. Conditions: 1 µM M^{pro} preincubated with 3 µM N3, 20 µM IPA-3 in the reaction buffer (20 mM HEPES, pH 7.5, 50 mM NaCl. The black spectrum is M^{pro} (33796 ± 1 Da) without an inhibitor. See Experimental Section for assay details. The structure of the inhibitor and a proposed outcome of reaction with a nucleophilic cysteine are shown.

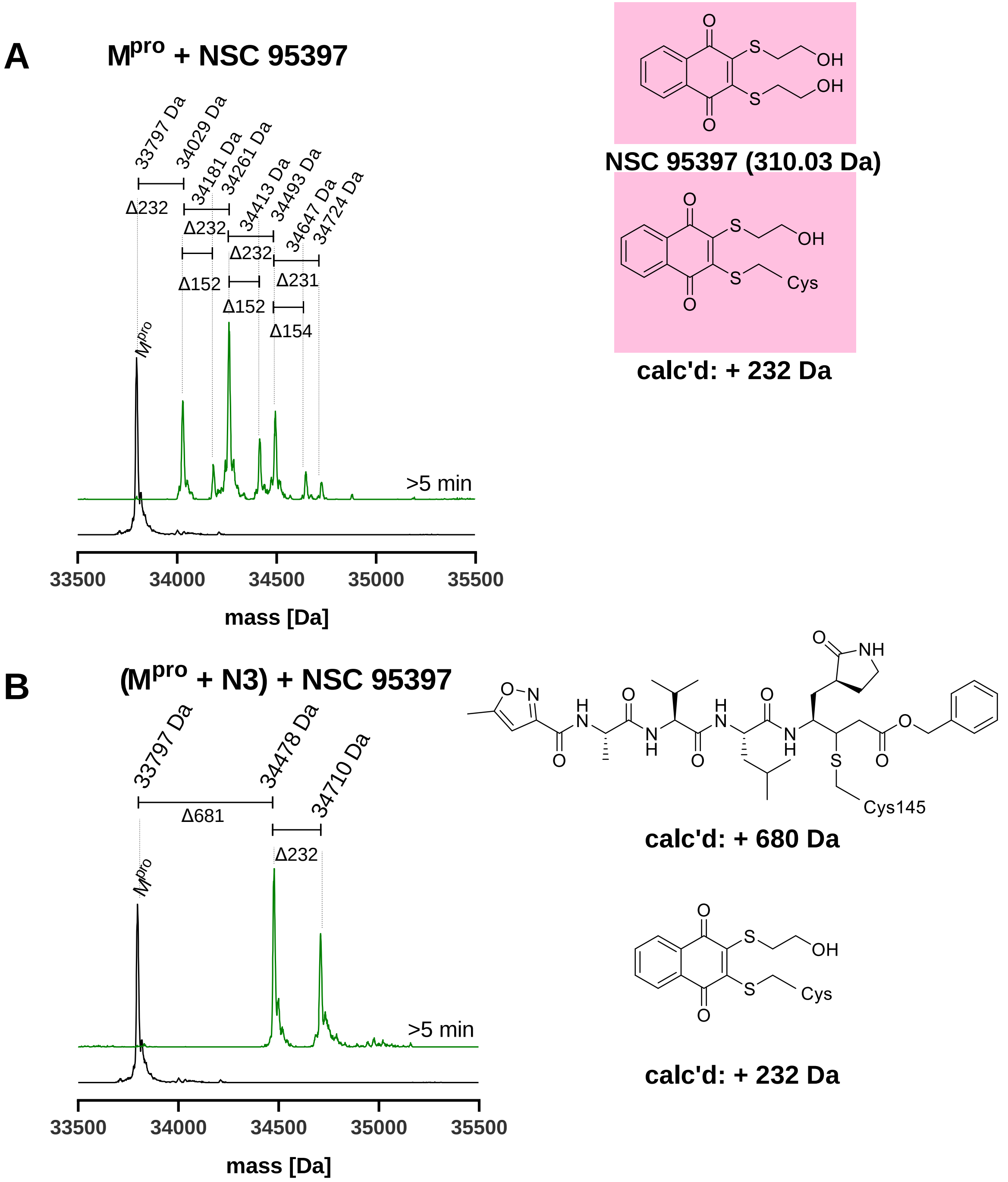

Figure S10. NSC 95397 reacts with M^{pro}. (**A**) NSC 95397 reacts with M^{pro} at multiple positions, possibly by Michael addition-elimination reaction. A 152 Da mass adduct may correspond to formation of disulfides with 2-mercaptoethanols. Conditions: 1 µM M^{pro}, 20 µM NSC 95397 in the reaction buffer (20 mM HEPES, pH 7.5, 50 mM NaCl). (**B**) NSC 95397 reacts with M^{pro} in the presence of inhibitor N3 suggesting NSC 95397 does not selectively bind to the M^{pro} active site Cys-145 residue. Conditions: 1 µM M^{pro} preincubated with 3 µM N3, 20 µM NSC 95397 in the reaction buffer (20 mM HEPES, pH 7.5, 50 mM NaCl). The black spectrum is M^{pro} (33796 ± 1 Da) without an inhibitor. See Experimental Section for assay details. The structure of the inhibitor and a proposed outcome of reaction with a nucleophilic cysteine are shown.

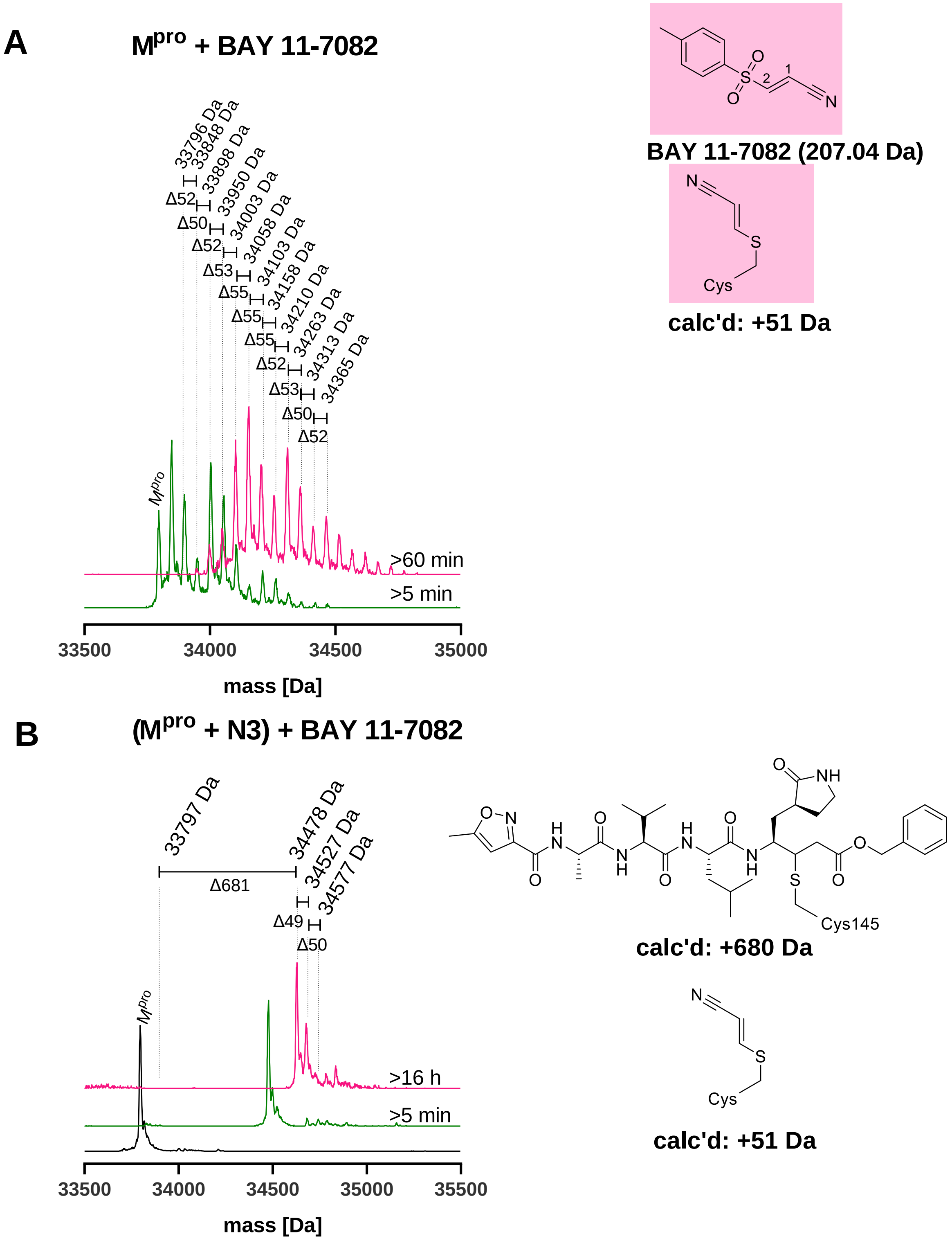

Figure S11. BAY 11-7082 reacts with M^{pro}. (**A**) BAY 11-7082 reacts with M^{pro} at multiple position, possibly by Michael addition of the α,β-unsaturated carbons at position 1 followed by an elimination reaction, although in a non-specific manner. Note that in contrast with our results, Michael reaction at the C2 positon of BAY 11-7082 is reported for tyrosine phosphatases inhibition.[23] Conditions: 1 µM M^{pro}, 20 µM BAY 11-7082 in the reaction buffer (20 mM HEPES, pH 7.5, 50 mM NaCl). (**B**) BAY 11-7082 (in part) reacts with M^{pro} in the presence of N3, although to a lesser extent compared to active site free M^{pro} suggesting it reacts at the active site. Conditions: 1 µM M^{pro} preincubated with 3 µM N3, 20 µM BAY 11-7082 in the reaction buffer (20 mM HEPES, pH 7.5, 50 mM NaCl).). The black spectrum is M^{pro} (33796 ± 1 Da) without an inhibitor. See Experimental Section for assay details. The structure of the inhibitor and a proposed outcome of reaction with a nucleophilic cysteine are shown.

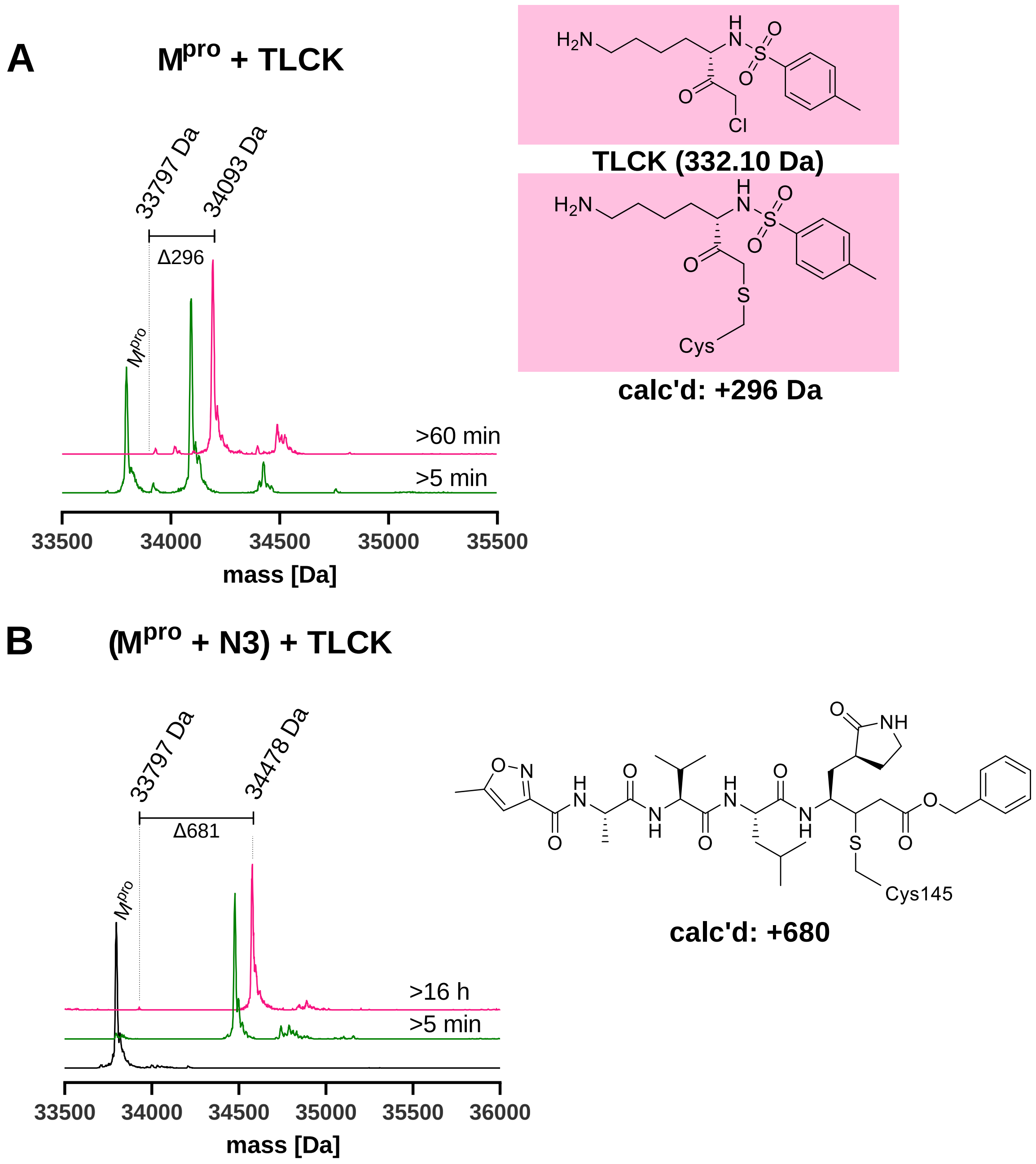

Figure S12. N_{α}-p-Toluenesulfonyl-*L*-lysine chloromethyl ketone (TLCK) covalently reacts with M^{pro}. (**A**) TLCK reacts with M^{pro}. Conditions: 1 µM M^{pro}, 20 µM TLCK in the reaction buffer (20 mM HEPES, pH 7.5, 50 mM NaCl). (**B**) M^{pro} which is preincubated with N3 to block the active site (**Figure S4**), does not react with TLCK. The results indicate that TLCK selectively binds to the M^{pro} active site. Conditions: 1 µM M^{pro} preincubated with 3 µM N3, 20 µM TLCK in the reaction buffer (20 mM HEPES, pH 7.5, 50 mM NaCl). The black spectrum is M^{pro} (33796 ± 1 Da) without an inhibitor. See Experimental Section for assay details. The structure of the inhibitor and a proposed outcome of reaction with a nucleophilic cysteine are shown.

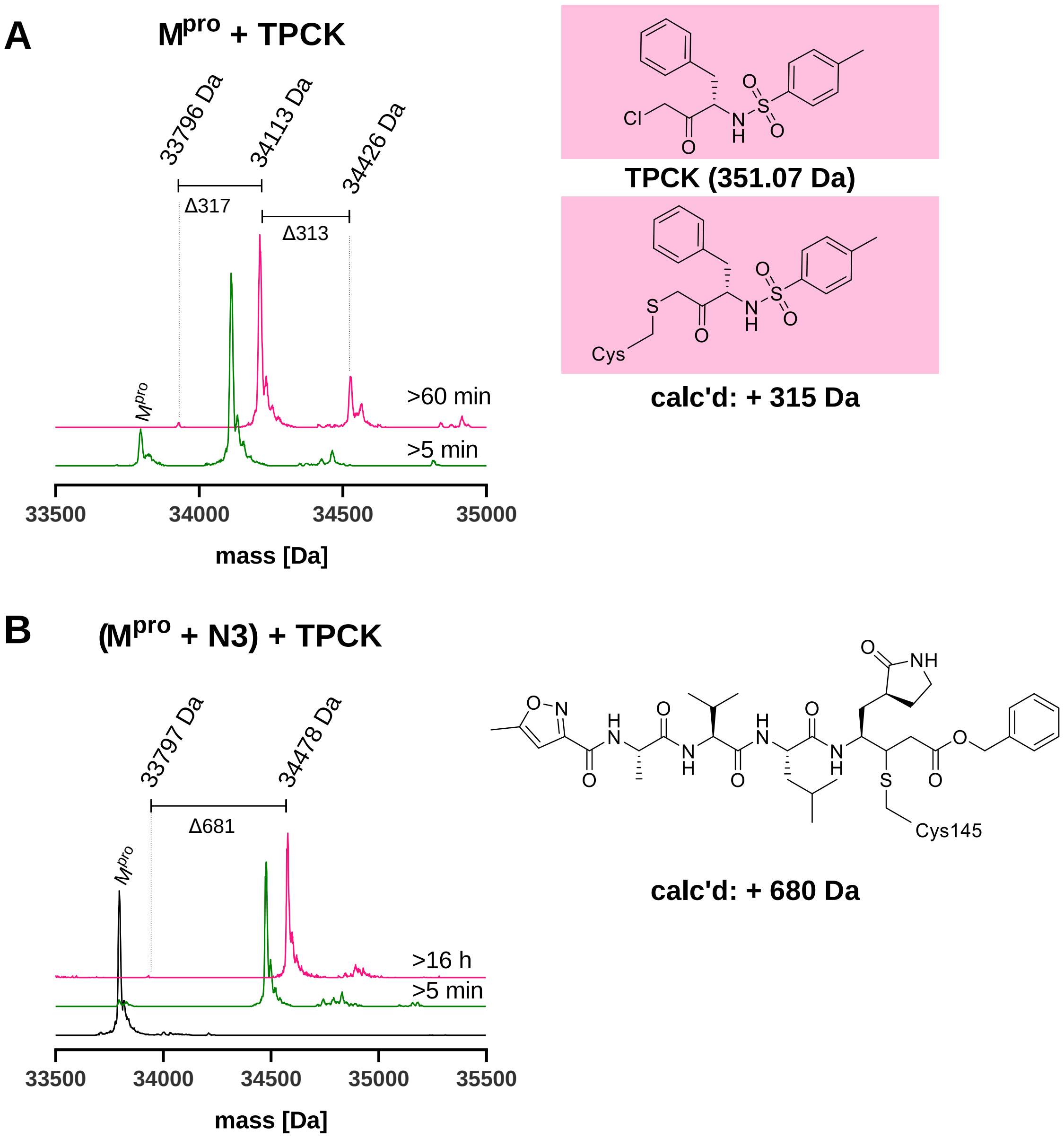

Figure S13. *N*-p-Toluenesulfonyl-L-phenylalanine chloromethyl ketone (TPCK) covalently reacts with M^{pro}. (**A**) TPCK reacts with M^{pro}**.** Conditions: 1 µM M^{pro}, 20 µM TPCK in the reaction buffer (20 mM HEPES, pH 7.5, 50 mM NaCl). (**B**) M^{pro} which is preincubated with N3 to block the active site Cys-145 (**Figure S4**) does not react with TPCK. The results indicate that TPCK selectively binds to the active site. Conditions: 1 µM M^{pro} preincubated with 3 µM N3, 20 µM TPCK in the reaction buffer (20 mM HEPES, pH 7.5, 50 mM NaCl). The black spectrum is M^{pro} (33796 ± 1 Da) without an inhibitor. See Experimental Section for assay details. The structure of the inhibitor and a proposed outcome of reaction with a nucleophilic cysteine are shown.

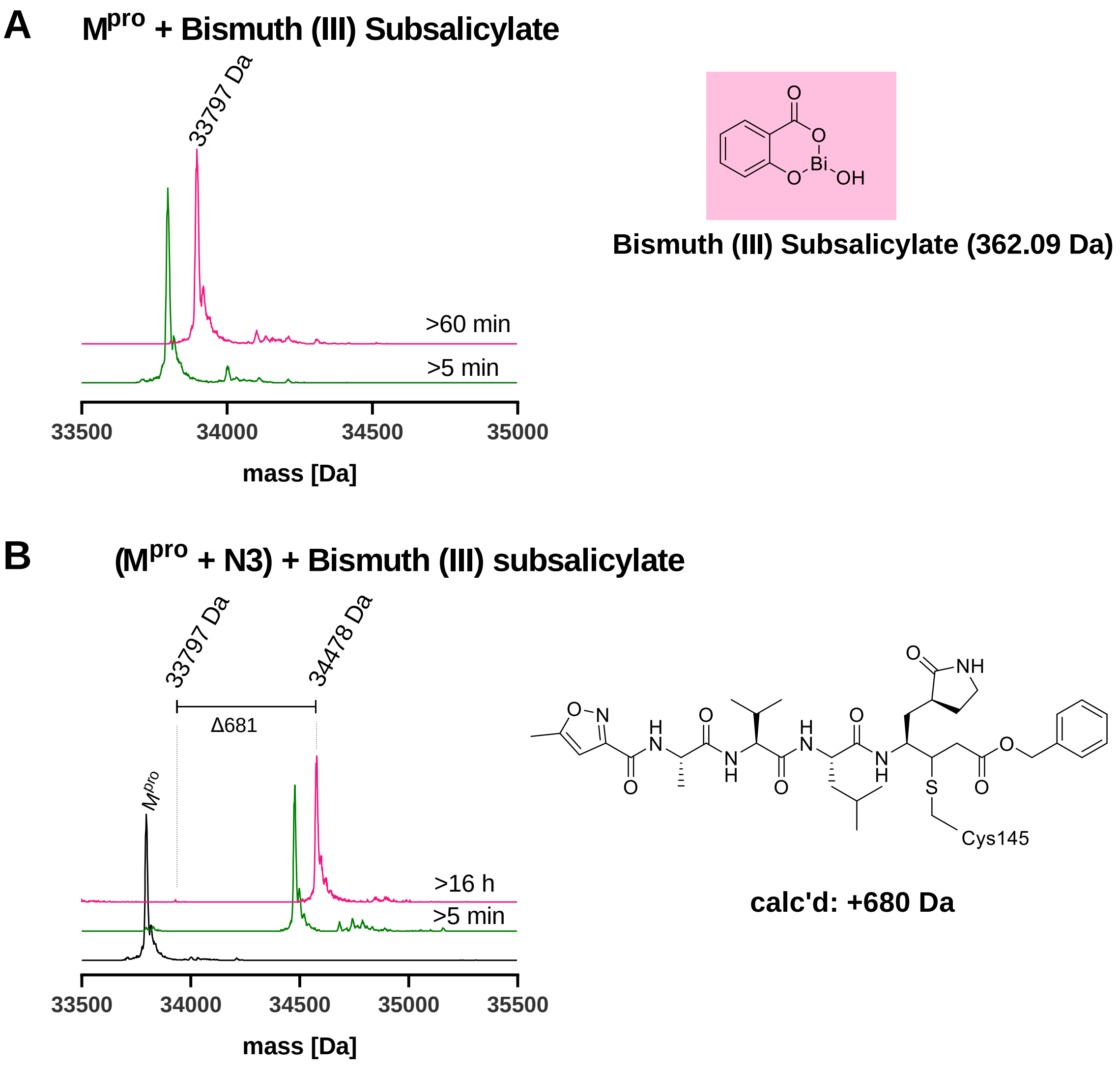

Figure S14. Bismuth(III) subsalicylate does not covalently react with M^{pro} to give a stable acyl-enzyme complex. (**A**) Bismuth(III) subsalicylate inhibits M^{pro} likely via non-covalent interaction, possibly involving metal ion co-ordination or thiol binding. Conditions: 1 µM M^{pro}, 20 µM bismuth(III) subsalicylate in the reaction buffer (20 mM HEPES, pH 7.5, 50 mM NaCl). (**B**) Bismuth(III) subsalicylate does not modify N3 reacted M^{pro}. Conditions: 1 µM M^{pro} preincubated with 3 µM N3, 20 µM bismuth(III) subsalicylate in the reaction buffer (20 mM HEPES, pH 7.5, 50 mM NaCl). The black spectrum is M^{pro} (33796 ± 1 Da) without an inhibitor. See Experimental Section for assay details. The structure of the inhibitor is shown.

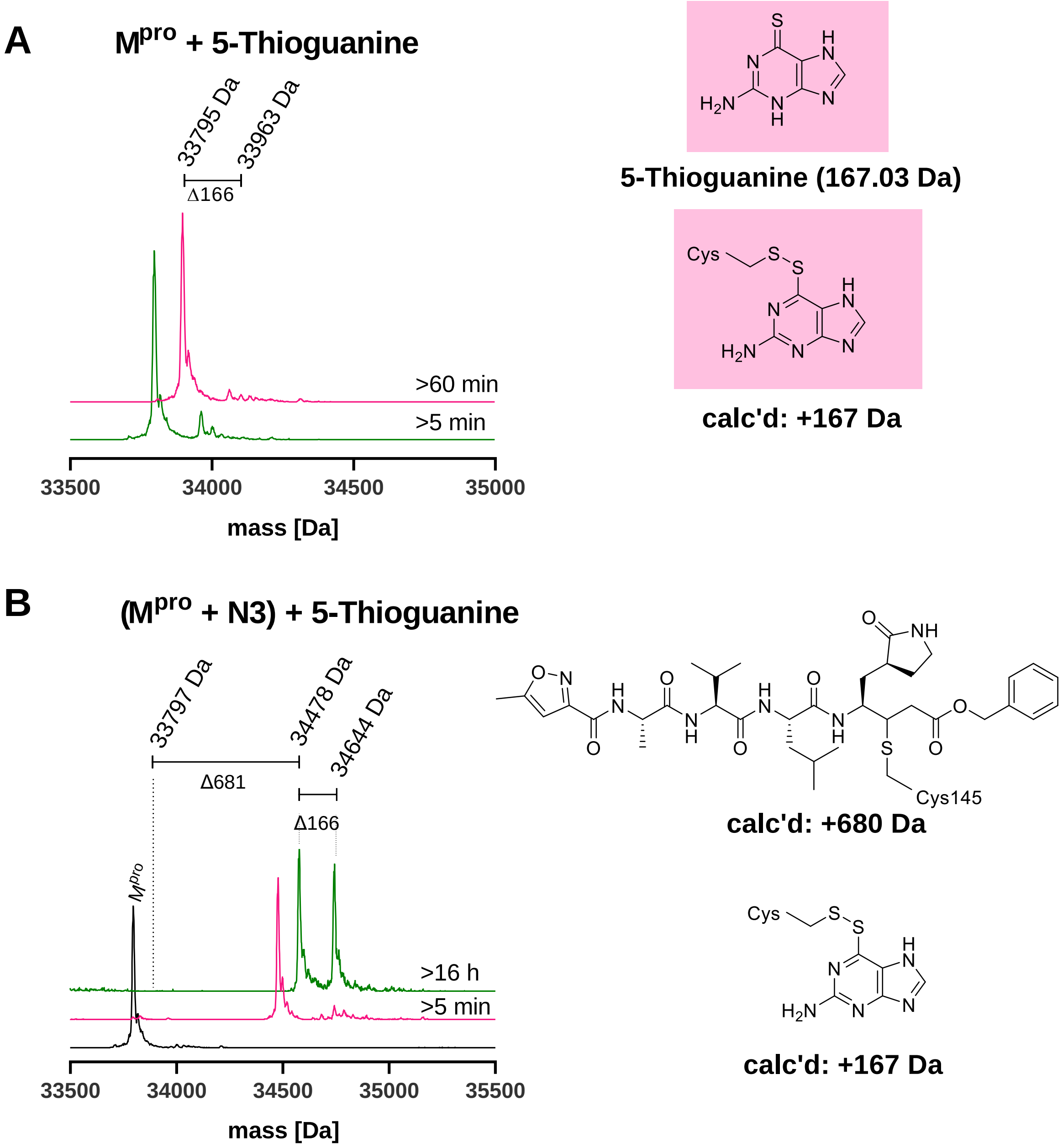

Figure S15. 5-Thioguanine does not covalently react with M^pro^ to give a stable acyl-enzyme complex. (**A**) 5-Thioguanine inhibits M^{pro} via non-covalent interactions. Conditions: 1 µM M^{pro}, 20 µM 5-thiogunaine in the reaction buffer (20 mM HEPES, pH 7.5, 50 mM NaCl). (**B**) 5-Thioguanine does not modify N3 reacted M^{pro}. A 166 Da adduct corresponding to the m/z difference of 5-thioguanine was observed after >16 h incubation with N3 reacted M^{pro} suggesting non-specific covalent labelling of M^{pro} cysteines by 5-thioguanine after prolonged incubation. Conditions: 1 µM M^{pro} preincubated with 3 µM N3, 20 µM 5-thioguanine in the reaction buffer (20 mM HEPES, pH 7.5, 50 mM NaCl). The black spectrum is M^{pro} (33796 ± 1 Da) without an inhibitor. See Experimental Section for assay details. The structure of the inhibitor and a proposed outcome of reaction with a nucleophilic cysteine are shown.

Table S5. Several β-lactam M^{pro} inhibitors identified from library screen (Figure S7). The observed inhibition of M^{pro} by cephalosporin C zinc salt is likely Zn(II) ion mediated(at least predominantly) (**Figure S16**).[24,25] See Experimental Section for assay details.

**LOPAC[1280]**	**% M^{pro} inhibition at 20 μM**
Cephalosporin C zinc salt	92.3
BLI-489 hydrate	77.3
Imipenem monohydrate	12.4
Cefaclor	8.7
**FDA APPROVED MOLECULES**	**% M^{pro} inhibition at 20 μM**
Cephalosporin C sodium	78.3
Ceftiofur hydrochloride	65.0
Cefprozil	57.5
Cefepime hydrochloride	20.6
Imipenem	12.4
Ceforanide	10.2
Cefalonium	9.2
Sulbactam	6.6

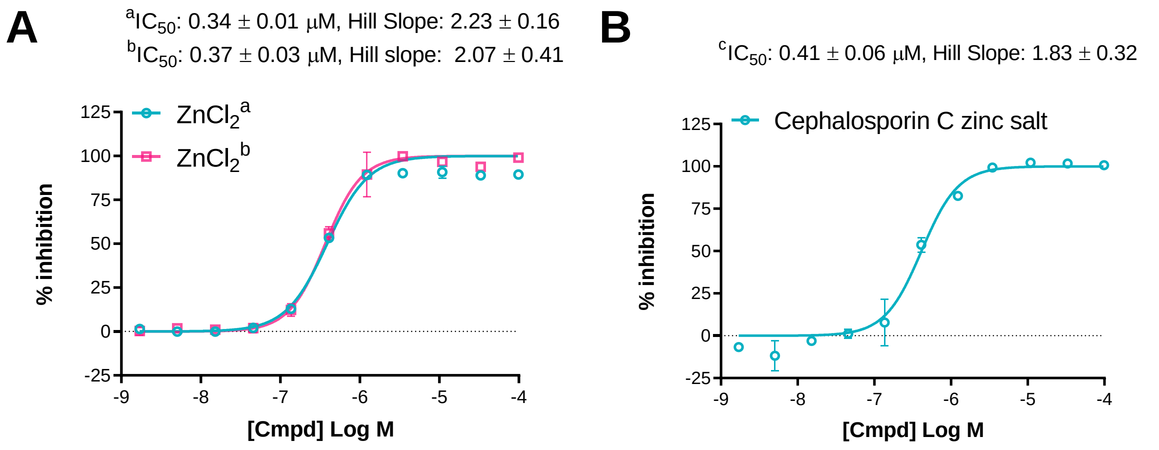

Figure S16. Zn ions inhibit M^{pro}. (**A**) Zinc ions inhibit M^{pro}, possibly by metal ion co-ordination of the His residues. 400 mM $ZnCl_2$ was prepared in 20 mM HCl and diluted to 20 mM in DMSO. M^{pro} was preincubated with $ZnCl_2$ for either (a) 30 min or (b) 60 min. Each data point represents the mean of technical duplicates (n=2 ± SD). (**B**) Much of the inhibition from cephalosporin C zinc salt is from Zn(II) ions. M^{pro} was preincubated with cephalosporin C zinc salt for (c) 22 minutes. IC_{50} values were calculated from the mean of the two technical duplicates (n=2 ± SD).

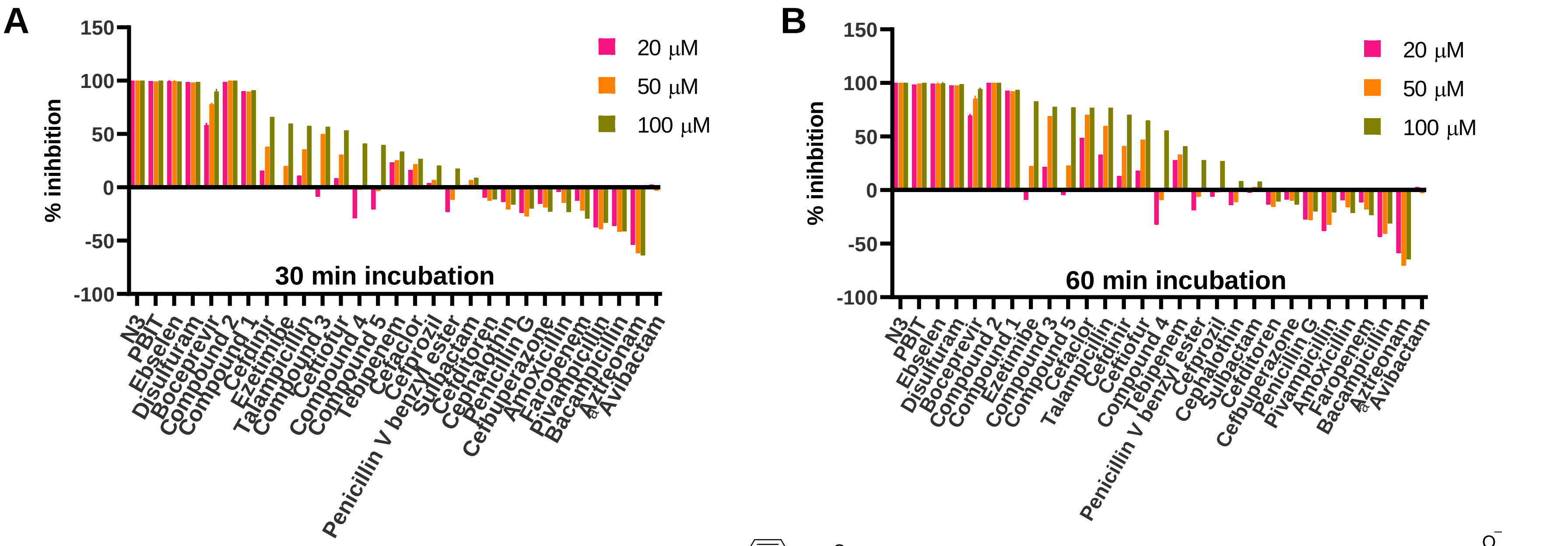

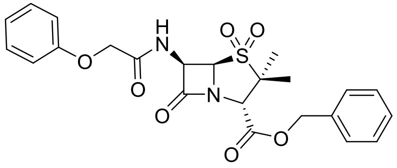

1

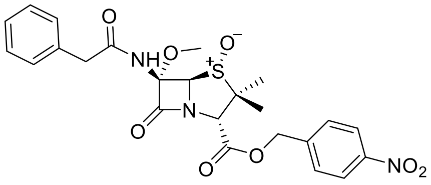

2

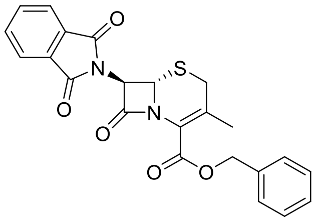

3

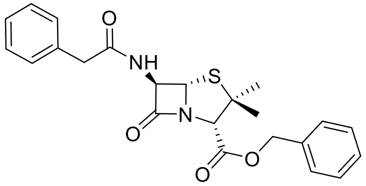

4

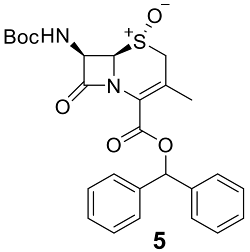

5

Figure S17. Assaying inhibition of M^pro^ by β-lactams (antibiotics). SPE-MS M^pro^ inhibition assays with β-lactams were performed at 20 μm (pink), 50 μm (orange) and 100 μM (green) single β-lactams concentrations with either (A) 30 min or (B) 60 min preincubation times. N3, PBIT, disulfiram and ebselen were used as positive inhibitor controls. Conditions: 0.15 μM M^pro^, 2.0 μM TSAVLQ/SGFRK-NH_2 peptide substrate in the reaction buffer (20 mM HEPES, pH 7.5, 50 mM NaCl). [a]Avibactam is a non-β-lactam β-lactamase inhibitor. **1** (penicillin V sulfone C3 benzyl ester) and **2** (C6-methoxy penicillin G sulfoxide C3 benzyl ester) were observed to manifest moderate inhibition. No inhibition was observed with penicillin G and penicillin V. Cefdinir, ceftiofur (cephalosporin), ezetimibe (monobactam), talampicillin (C3 protected penicillin), **3** (C6 epimer of cephalosporin benzyl ester), **4** (penicillin V C5 epimer benzyl ester) and **5** (cephalosporin sulfoxide diphenyl ester) exhibited weak time dependent inhibition.

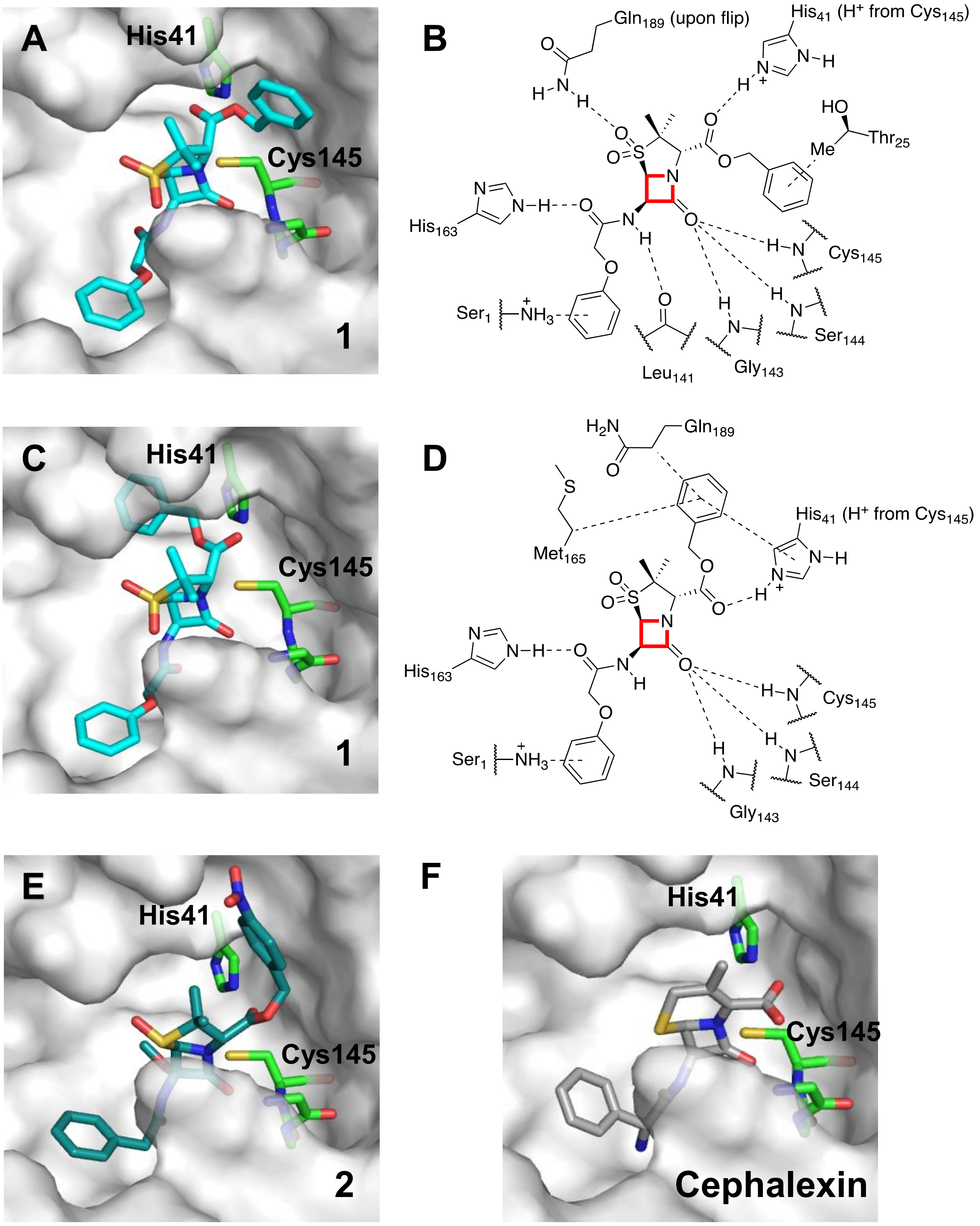

Figure S18. Docking poses of β-lactams 1, 2, and cephalexin in the M^{pro} active site based on a reported crystal structure (PDB: 6LU7).[1] For **1**, two alternative poses that may enable reaction with Cys-145 are presented (**A** and **C**), with key potential interactions shown in **B** and **D**, respectively. In both cases, the β-lactam carbonyl oxygens are positioned to hydrogen bond with the backbone amides of Gly-143, Ser-144 and Cys-145, as does the carbonyl oxygen of the scissile substrate amide. The C6 amide side chains adopt an extended conformation along the P1 pocket that allows interactions with His-163 and Ser-1 (the latter being of the other chain of the M^{pro} dimer). The two poses differ in the positioning of the C3 side chain, in which the benzyl group could interact hydrophobically either with Thr-25 (**B**) or fit into the P2 pocket (**D**). The β-lactam core of **2** (**E**) and cephalosporins such as cephalexin (**F**) could bind in the M^{pro} active site in a manner similar to that of **1**. The β-lactam containing compounds were docked using AutoDock Vina (1.1.2)[26] in a cubic box of side length 3 nm centred at the Cys-145 sulfur atom. Interactions were identified by visual inspection and the Protein-Ligand Interaction Profiler.[27] Note that no M^{pro} inhibition was observed with cephalexin (**Figure S21 I**).

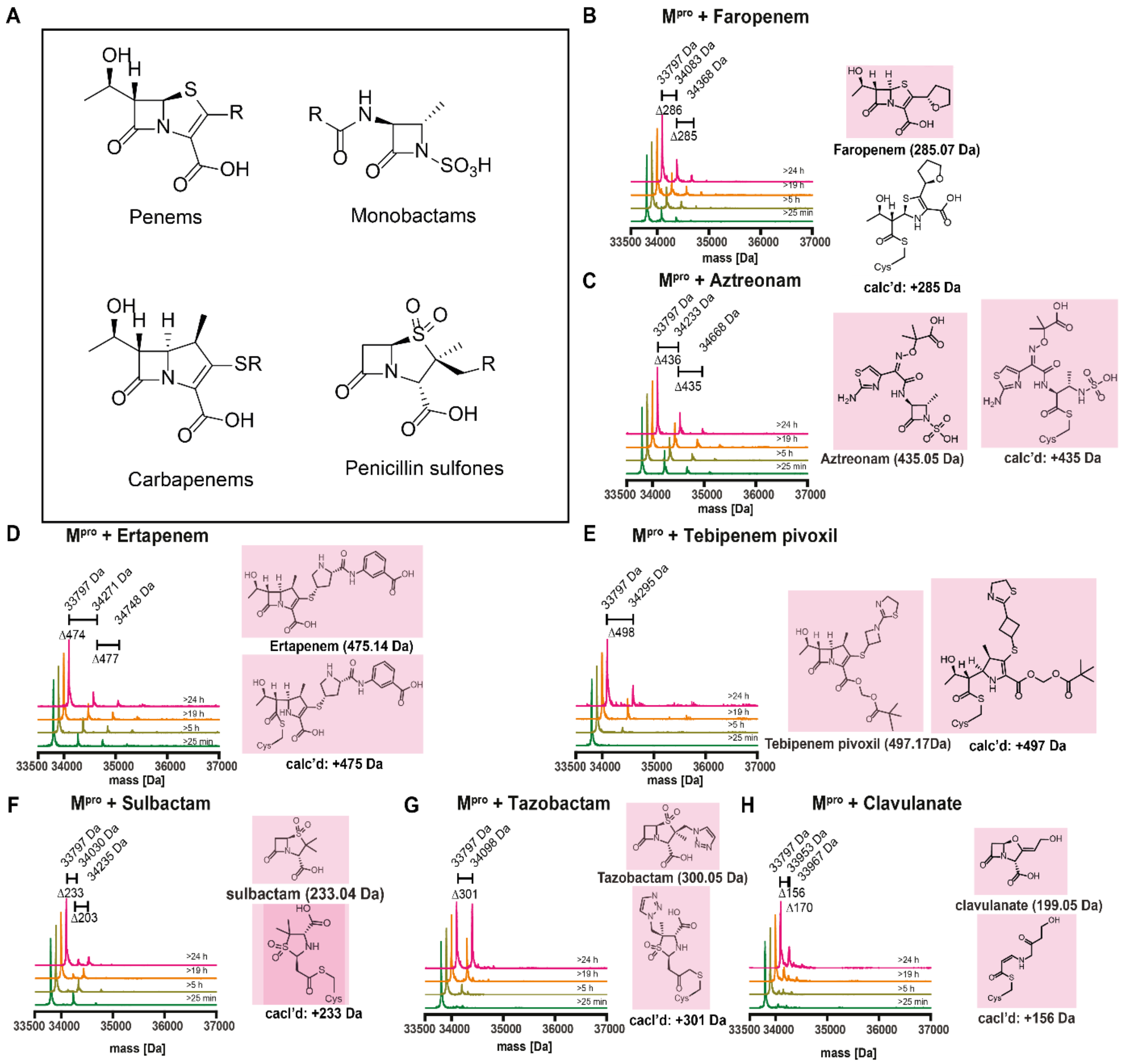

Figure S19. Some β-lactams react with Mpro. (**A**) Different classes of β-lactams investigated. Mpro (1 µM) was incubated with a 100 fold excess of: (**B**) faropenem (penem), (**C**) aztreonam (monobactam), (**D**) ertapenem (carbapenem), (**E**) tebipenem (C3 protected carbapenem prodrug), (**F**) sulbactam, (**G**) tazobactam (penicillin sulfones), or (**H**) clavulanate (penicillin sulfones) for (at least) 25 min, 5 h, 9 h and 24 h in the reaction buffer (20 mM HEPES, pH 7.5, 50 mM NaCl). Relatively low levels of acylation were observed for all β-lactams with the exception of tazobactam for which the extent of apparent acylation increased over time. See Experimental Section for assay details. The structureS of the inhibitors and the possible outcomes of reaction with a nucleophilic cysteine are shown.

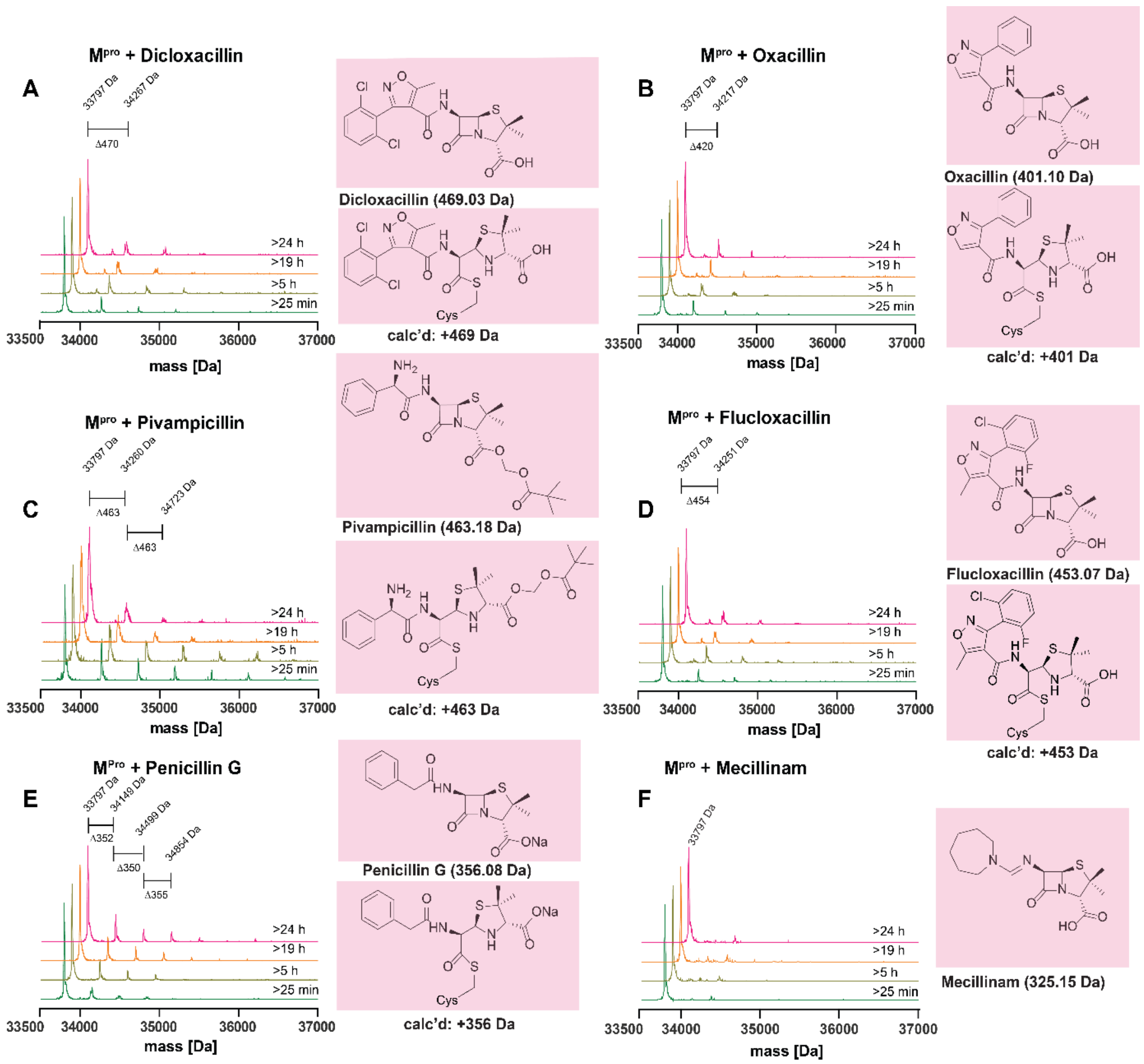

Figure S20. Several penicillins react with M^pro^. M^pro^ (1 µM) was incubated with a 100 fold excess of: (**A**) dicloxacillin, (**B**) oxacillin, (**C**) pivampicillin, (**D**) flucloxacillin, (**E**) peniclin G, or (**F**) mecillinam for (at least) 25 min, 5 h, 9 h and 24 h in the reaction buffer (20 mM HEPES, pH 7.5, 50 mM NaCl). All the penicillins investigated apparently covalently modified M^pro^ with the exception of mecillinam. In no case was M^pro^ modification complete**.** The mass discrepancy of +19 Da adduct for M^pro^ incubated with oxacillin may correspond to a salt adduct or hydration. See Experimental Section for assay details. The structures of the inhibitors and possible outcomes of reaction with a nucleophilic cysteine are shown.

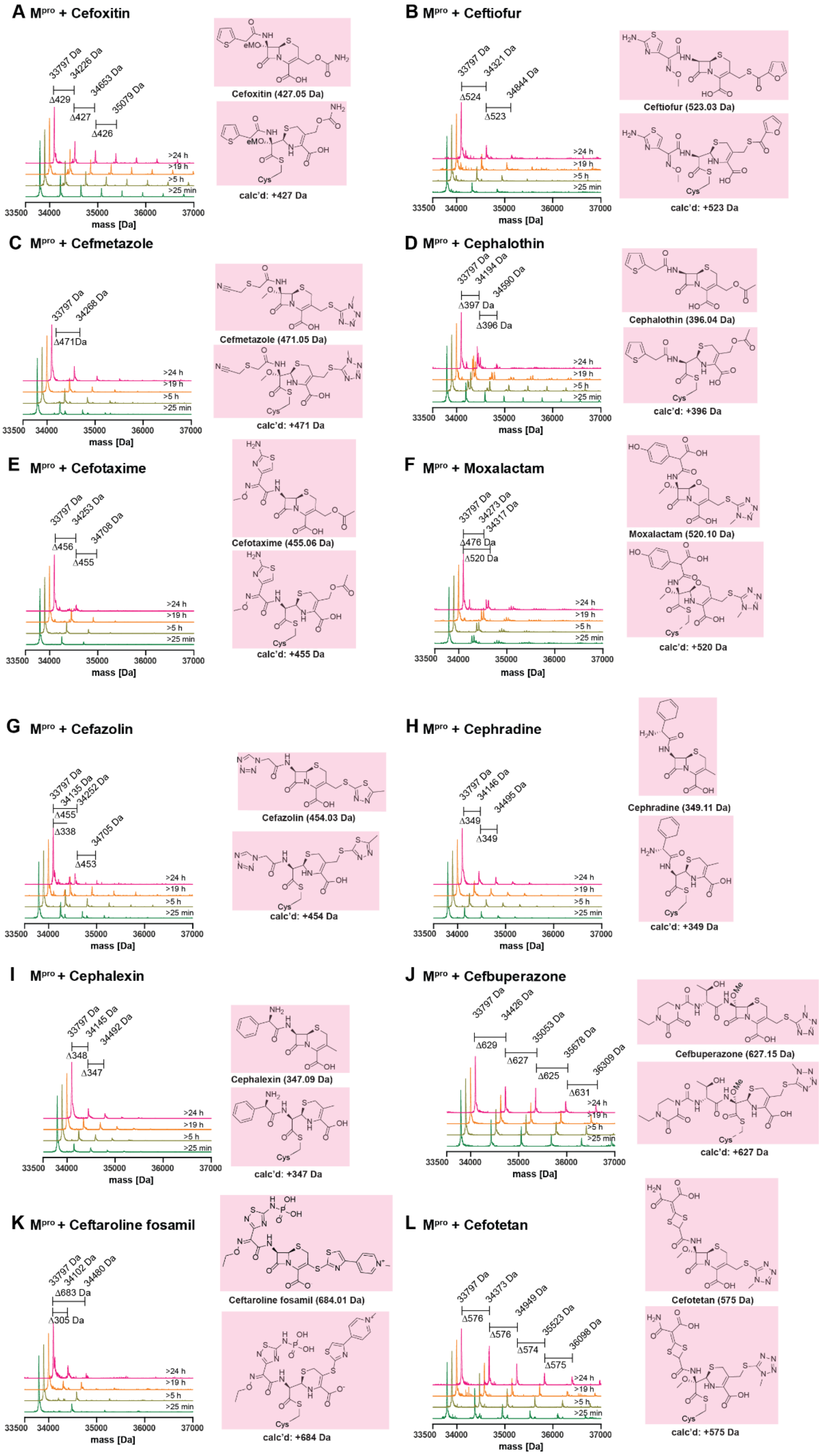
A Mpro + Cefoxitin
Cefoxitin (427.05 Da)
calc'd: +427 Da
B Mpro + Ceftiofur
Ceftiofur (523.03 Da)
calc'd: +523 Da
C Mpro + Cefmetazole
Cefmetazole (471.05 Da)
calc'd: +471 Da
D Mpro + Cephalothin
Cephalothin (396.04 Da)
calc'd: +396 Da
E Mpro + Cefotaxime
Cefotaxime (455.06 Da)
calc'd: +455 Da
F Mpro + Moxalactam
Moxalactam (520.10 Da)
calc'd: +520 Da
G Mpro + Cefazolin
Cefazolin (454.03 Da)
calc'd: +454 Da
H Mpro + Cephradine
Cephradine (349.11 Da)
calc'd: +349 Da
I Mpro + Cephalexin
Cephalexin (347.09 Da)
calc'd: +347 Da
J Mpro + Cefbuperazone
Cefbuperazone (627.15 Da)
calc'd: +627 Da
K Mpro + Ceftaroline fosamil
Ceftaroline fosamil (684.01 Da)
calc'd: +684 Da
L Mpro + Cefotetan
Cefotetan (575 Da)
calc'd: +575 Da
>24 h
>19 h
>5 h
>25 min
mass [Da]
Cys

Figure S21. Several cephalosporins react with M^{pro}. M^{pro} (1 µM) was incubated with a 100 fold excess of: (**A**) cefoxitin, (**B**) ceftiofur, (**C**) cefmetazole, (**D**) cephalothin, (**E**) cefotaxime, (**F**) moxalactam, (**G**) cefazolin, (**H**) cephradine, (**I**) cephalexin, (**J**) cefbuperazone, (**K**) ceftaroline fosoamil, or (**L**) cefotetan for (at least) 25 min, 5 h, 9 h and 24 h in the reaction buffer (20 mM HEPES, pH 7.5, 50 mM NaCl). For cefotetan +576 Da adducts, cefbuperazone +627 Da adducts and cefoxitin +427 Da adducts, evidence for more than one reaction was accrued. For cefazolin, an additional +338 Da adduct was observed. For M^{pro} incubated with moxalactam, an additional peak with mass shift (+476 Da) corresponding to -45 Da loss relative to the moxalactam adduct (+520 Da) was observed, possibly due to decarboxylation of moxalactam. as reported in MS analysis of carbapenems[28] and other β-lactams[29]. The structures of the inhibitors and possible outcomes of reaction with a nucleophilic cysteine are shown. Refer to Experimental Section for assay details.

References

1. Z. Jin, X. Du, Y. Xu, Y. Deng, M. Liu, Y. Zhao, B. Zhang, X. Li, L. Zhang, C. Peng, Y. Duan, J. Yu, L. Wang, K. Yang, F. Liu, R. Jiang, X. Yang, T. You, X. Liu, X. Yang, F. Bai, H. Liu, X. Liu, L. W. Guddat, W. Xu, G. Xiao, C. Qin, Z. Shi, H. Jiang, Z. Rao and H. Yang, *Nature*, 2020, **582**, 289-293.
2. X. Xue, H. Yang, W. Shen, Q. Zhao, J. Li, K. Yang, C. Chen, Y. Jin, M. Bartlam and Z. Rao, *J. Mol. Biol.*, 2007, **366**, 965-975.
3. C. V. Robinson, T. J. El-Baba, C. A. Lutomski, A. L. Kantsadi, T. R. Malla, T. John, V. Mikhailov, J. R. Bolla, C. J. Schofield, N. Zitzmann and I. Vakonakis, *Angew. Chem. Int. Ed. Engl.*, 2020, **n/a**.
4. S. K. Bharti and R. Roy, *Trends Analyt. Chem.*, 2012, **35**, 5-26.
5. A. W. Chow, J. R. E. Hoover and N. M. Hall, *J. Org. Chem.*, 1962, **27**, 1381-&.
6. *Author, USA Pat.,* FR 2207695, 1972.
7. T. Fekner, J. E. Baldwin, R. M. Adlington, T. W. Jones, C. K. Prout and C. J. Schofield, *Tetrahedron*, 2000, **56**, 6053-6074.
8. R. Busson and H. Vanderhaeghe, *J. Org. Chem.*, 1976, **41**, 2561-2565.
9. J. E. Baldwin, R. M. Adlington, R. T. Aplin, N. P. Crouch and R. Wilkinson, *Tetrahedron*, 1992, **48**, 6853-6862.
10. L. Zhang, D. Lin, X. Sun, U. Curth, C. Drosten, L. Sauerhering, S. Becker, K. Rox and R. Hilgenfeld, *Science*, 2020, **368**, 409-412.
11. C. Ceraolo and F. M. Giorgi, *J. Med. Virol.*, 2020, **92**, 522-528.
12. J. E. Blanchard, N. H. Elowe, C. Huitema, P. D. Fortin, J. D. Cechetto, L. D. Eltis and E. D. Brown, *Chem. Biol.*, 2004, **11**, 1445-1453.
13. K. Fan, P. Wei, Q. Feng, S. Chen, C. Huang, L. Ma, B. Lai, J. Pei, Y. Liu, J. Chen and L. Lai, *J. Biol. Chem.*, 2004, **279**, 1637-1642.
14. U. Bacha, J. Barrila, A. Velazquez-Campoy, S. A. Leavitt and E. Freire, *Biochemistry*, 2004, **43**, 4906-4912.
15. R. Y. Kao, A. P. To, L. W. Ng, W. H. Tsui, T. S. Lee, H. W. Tsoi and K. Y. Yuen, *FEBS Lett.*, 2004, **576**, 325-330.
16. W. Vuong, M. B. Khan, C. Fischer, E. Arutyunova, T. Lamer, J. Shields, H. A. Saffran, R. T. McKay, M. J. van Belkum, M. A. Joyce, H. S. Young, D. L. Tyrrell, J. C. Vederas and M. J. Lemieux, *Nat. Commun.*, 2020, **11**, 4282.
17. V. Graziano, W. J. McGrath, A. M. DeGruccio, J. J. Dunn and W. F. Mangel, *FEBS Lett.*, 2006, **580**, 2577-2583.
18. J. H. Zhang, T. D. Chung and K. R. Oldenburg, *J. Biomol. Screen.*, 1999, **4**, 67-73.
19. Z. He, W. Zhao, W. Niu, X. Gao, X. Gao, Y. Gong and X. Gao, *bioRxiv*, 2020, DOI: 10.1101/2020.05.28.120642, 2020.2005.2028.120642-122020.120605.120628.120642.
20. S. W. Deacon, A. Beeser, J. A. Fukui, U. E. Rennefahrt, C. Myers, J. Chernoff and J. R. Peterson, *Chem. Biol.*, 2008, **15**, 322-331.
21. M. Itokazu, T. Matsunaga and Y. Oshita, *Clin. Ther.*, 1995, **17**, 60-73.
22. E. Shaw, in *Enzyme Structure*, Academic Press, 1967, vol. 11, pp. 677-686.
23. N. Krishnan, G. Bencze, P. Cohen and N. K. Tonks, *FEBS J.*, 2013, **280**, 2830-2841.
24. K. J. Seamon and J. T. Stivers, *J. Biomol. Screen.*, 2015, **20**, 801-809.
25. L. Brewitz, A. Tumber, I. Pfeffer, M. A. McDonough and C. J. Schofield, *Sci. Rep.*, 2020, **10**, 8650.
26. O. Trott and A. J. Olson, *J. Comput. Chem.*, 2010, **31**, 455-461.
27. S. Salentin, S. Schreiber, V. J. Haupt, M. F. Adasme and M. Schroeder, *Nucleic Acids Res.*, 2015, **43**, W443-447.
28. E. M. Steiner, G. Schneider and R. Schnell, *FEBS J.*, 2017, **284**, 725-741.
29. R. P. A. Brown, R. T. Aplin and C. J. Schofield, *Biochemistry*, 1996, **35**, 12421-12432
